# Mitochondria-targeted and ultrasound-responsive nanoparticles for oxygen and nitric oxide codelivery to reverse immunosuppression and enhance sonodynamic therapy for immune activation

**DOI:** 10.7150/thno.62572

**Published:** 2021-07-25

**Authors:** Changwei Ji, Jingxing Si, Yan Xu, Wenjing Zhang, Yaqian Yang, Xin He, Hao Xu, Xiaozhou Mou, Hao Ren, Hongqian Guo

**Affiliations:** 1 Department of Urology, Drum Tower Hospital, Medical school of Nanjing University, Institute of Urology, Nanjing University, Nanjing 210008, Jiangsu, China.; 2 Key Laboratory of Tumor Molecular Diagnosis and Individualized Medicine of Zhejiang Province, Zhejiang Provincial People's Hospital, People's Hospital of Hangzhou Medical College, Hangzhou 310014, Zhejiang, China.; 3 Clinical Research Institute, Zhejiang Provincial People's Hospital, People's Hospital of Hangzhou Medical College, Hangzhou 310014, Zhejiang, China.; 4 School of Pharmaceutical Science, Nanjing Tech University, Nanjing 211816, Jiangsu, China.

**Keywords:** Mitochondria-targeted, Oxygen and nitric oxide codelivery, Reverse immunosuppression, Sonodynamic therapy, Immune response

## Abstract

**Background**: Sonodynamic therapy (SDT) is a promising strategy to inhibit tumor growth and activate antitumor immune responses for immunotherapy. However, the hypoxic and immunosuppressive tumor microenvironment limits its therapeutic efficacy and suppresses immune response.

**Methods:** In this study, mitochondria-targeted and ultrasound-responsive nanoparticles were developed to co-deliver oxygen (O_2_) and nitric oxide (NO) to enhance SDT and immune response. This system (PIH-NO) was constructed with a human serum albumin-based NO donor (HSA-NO) to encapsulate perfluorodecalin (FDC) and the sonosensitizer (IR780).* In vitro*, the burst release of O_2_ and NO with US treatment to generate reactive oxygen species (ROS), the mitochondria targeting properties and mitochondrial dysfunction were evaluated in tumor cells. Moreover, *in vivo*, tumor accumulation, therapeutic efficacy, the immunosuppressive tumor microenvironment, immunogenic cell death, and immune activation after PIH-NO treatment were also studied in 4T1 tumor bearing mice.

**Results:** PIH-NO could accumulate in the mitochondria and relive hypoxia. After US irradiation, O_2_ and NO displayed burst release to enhance SDT, generated strongly oxidizing peroxynitrite anions, and led to mitochondrial dysfunction. The release of NO increased blood perfusion and enhanced the accumulation of the formed nanoparticles. Owing to O_2_ and NO release with US, PIH-NO enhanced SDT to inhibit tumor growth and amplify immunogenic cell death *in vitro* and* in vivo*. Additionally, PIH-NO promoted the maturation of dendritic cells and increased the number of infiltrating immune cells. More importantly, PIH-NO polarized M2 macrophages into M1 phenotype and depleted myeloid-derived suppressor cells to reverse immunosuppression and enhance immune response.

**Conclusion:** Our findings provide a simple strategy to co-deliver O_2_ and NO to enhance SDT and reverse immunosuppression, leading to an increase in the immune response for cancer immunotherapy.

## Introduction

Sonodynamic therapy (SDT) is a promising therapeutic modality for cancer because of the minimally invasive deep tissue penetration of ultrasound (US) 1, 2. SDT applies sonosensitizers that are activated by US to transfer energy to the surrounding oxygen (O_2_), generating reactive oxygen species (ROS) to directly kill or induce apoptosis of tumor cells 3. Recent reports have suggested that SDT can expose calreticulin (CRT) and release high mobility group box 1 (HMGB-1) in dying tumor cells to induce immunogenic cell death (ICD), which promotes the maturation of dendritic cells (DCs) and provokes an antitumor immune response 4, 5. The extent of ICD effect is highly dependent on the therapeutic efficacy of SDT. However, solid tumors are characterized by hypoxia due to limited O_2_ diffusion and insufficient blood perfusion, which severely restricts the effectiveness and clinical applications of SDT 6, 7. In addition, the consumption of O_2_ during the SDT process may further aggravate tumor hypoxia 8, 9. To overcome this limitation, various strategies have been reported to alleviate hypoxia, such as delivering O_2_ to tumor sites with certain oxygen-carriers, generating O_2_ from endogenous hydrogen peroxide *in situ* with catalase or inhibiting inspiration to decrease O_2_ consumption 10-12. Among these, perfluorocarbon (PFC) has been widely applied as an artificial blood substitute because of its high oxygen-carrying capacity and biocompatibility 13, 14. It has been reported that US could trigger O_2_ burst release from PFC to remarkably enhance both radiation and photodynamic therapy 15, 16. Therefore, burst-like oxygen release with US could significantly relieve hypoxia and increase ROS generation to improve SDT, thus amplifying ICD to enhance the immune response.

Based on previous studies, abundant tumor-associated macrophages (TAMs) and myeloid-derived suppressor cells (MDSCs), two critical drivers of the immunosuppressive tumor microenvironment (TME), suppress T-cell activation and proliferation to restrict the immune response of SDT 17, 18. In the tumor site, TAMs are usually polarized to an activated M2 phenotype, which impairs the presentation of antigens to suppress the immune response 19, 20. In contrast, M1 macrophages are extremely potent effector cells that inhibit tumor growth by releasing proinflammatory factors 21. Therefore, depleting MDSCs and the polarization of M2 macrophages to the M1 phenotype would be an attractive approach to reverse the immunosuppressive TME and thus enhance the SDT-induced immune response for immunotherapy.

Nitric oxide (NO) has been widely applied as a gas therapy, as it can directly kill cancer cells and act as a regulator to modulate the immunosuppressive TME 22-24. NO can inhibit tumor respiration and normalize tumor blood vessels to increase blood perfusion and overcome tumor hypoxia, reeducating M2 to M1 and decreasing the percentage of MDSCs 25-27. In addition, recent studies have found that NO can react with the ROS generated from SDT or photodynamic therapy to produce more powerful oxidant reactive nitrogen species (RNS), especially peroxynitrite anions (ONOO-), which can effectively inhibit tumor growth 28, 29. More importantly, the generation of RNS could also polarize M2 to M1 macrophages and deplete MDSCs to reverse the immunosuppressive TME and enhance tumor immunotherapy 30. However, NO is unstable and its delivery and release at the tumor site is difficult 31. In our previous study, near-infrared (NIR) light was applied to trigger NO release to combine photothermal therapy with gas therapy 32. However, the penetration depth of NIR light is limited, which restricts the therapeutic efficacy of this combination. Previous studies have reported that US can trigger NO donors to release NO and enhance SDT 33, 34. Therefore, we hypothesize that during SDT, US is an effective way to trigger NO release and thus reverse the immunosuppressive TME and provoke an anti-tumor immune response. However, the short lifetimes and diffusion ranges of ROS and NO limit their therapeutic efficacy and immune responses 35, 36. It is therefore important to perform SDT with NO release by selective accumulation in subcellular organelles to enhance therapeutic efficacy.

**Figure 1:**
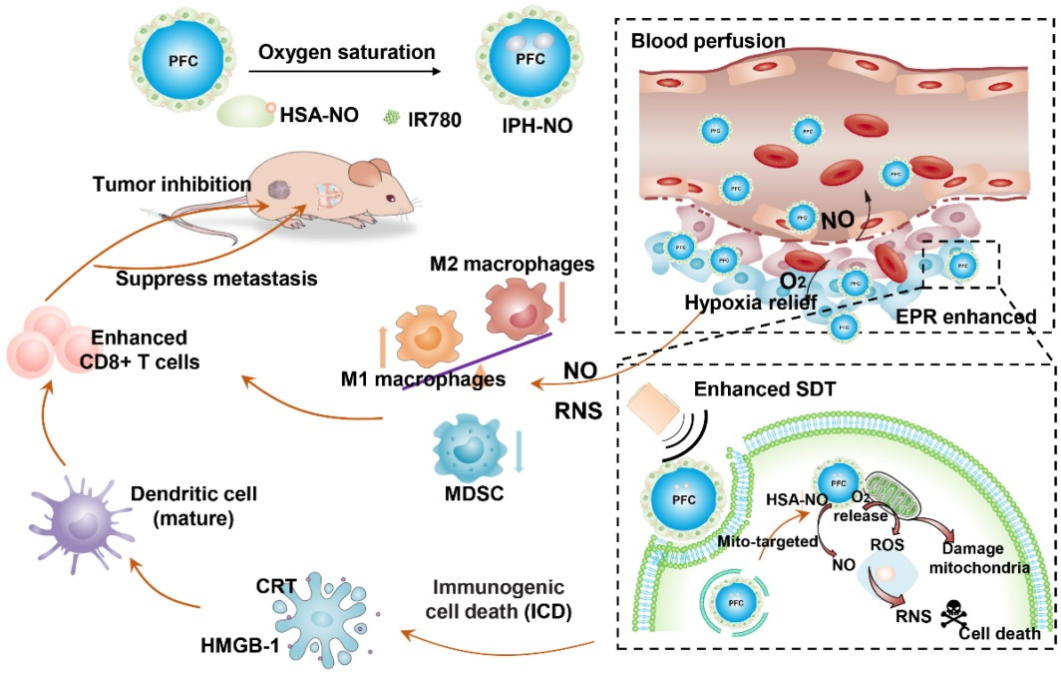
Schematic illustration of PIH-NO for cancer therapy and immune activation. PIH-NO was constructed by IR780 and HSA-NO with oxygen saturation. After intravenous injection, it could increase blood perfusion, increase EPR effect and relive hypoxia. It could prefer to accumulate in mitochondria. After with ultrasound irradiation, it could release oxygen and NO to enhance SDT, and damage mitochondria, which induce immunogenic cell death to promote DC maturation. The generation NO and RNS could polarize M2 macrophages to M1 phenotype and reduce MDSC to reverse immunosuppression microenvironment. All these approaches increase CD8^+^ T cells infiltration to inhibit primary tumor and lung metastasis.

Mitochondria play a critical role in energy production and cell apoptosis 37. Stimulation of and an imbalance in ROS in the mitochondria could lead to a loss of mitochondrial membrane and increased mitochondrial membrane permeability. These features damage mitochondrial functions to activate the intrinsic pathway of apoptosis 38-40. Therefore, in this study, we developed nanoparticles with mitochondria-targeted properties to co-deliver NO and O_2_ to enhance SDT, reverse immunosuppressive TME and increase anti-tumor immune response. PIH-NO was constructed with an human serum albumin-based NO donor (HSA-NO) as the carrier to encapsulate perfluorodecalin (FDC) and the IR780 41. FDC is a type of PFC with great biocompatibility, which has been approved as artificial blood substitute due to its high oxygen-carrying capacity 42. Additionally, in our previous, HSA-NO has been used as a NO donor and drug carrier, which is responsive to US and glutathione for NO release 32. IR780 serves as sonosensitizer for SDT. As shown in Figure 1, after intravenous injection into tumor-bearing mice, the generation of NO from PIH-NO increased blood perfusion to enhance accumulation. The PIH-NO accumulated in the mitochondria because IR780 is preferentially retained in mitochondria 43, 44. Upon US irradiation, PIH-NO produced a burst-like release of O_2_ and NO to relieve hypoxia, which increased ROS and ONOO- generation to enhance the therapeutic efficacy of SDT *in vivo* and *in vitro*. The generation of O_2_ and NO from PIH-NO at the tumor site reeducated M2 to M1 macrophages and reduced the number of MDSCs to reverse the immunosuppressive TME. In addition, O_2_-enhanced SDT in combination with NO therapy amplified ICD to promote DC maturation and increase the number of infiltrating immune cells for enhanced immunotherapy. Our study provides a promising strategy to deliver gases to enhance SDT and reverse the immunosuppressive TME for cancer immunotherapy.

## Experimental Section

### Materials

IR780 was purchased from Sigma Corporation. Griess assay, H_2_DCFDA, Mitotracker Red and DAF-FM DA were obtained by Beyotime Institute of Biotechnology (China). Singlet Oxygen Sensor Green (SOSG) was purchased from Thermo Fisher Scientific. Cytotoxicity ROS-ID Hypoxia/Oxidative Stress Detection Kit was obtained from Enzo Life Sciences. The 2-iminothiolane, isopentylnitrite, perfluorodecalin (FDC) and fluorescein isothiocyanate (FITC) were obtained from Aladdin Industrial Corporation (Shanghai, China). Mitotracker Green, Mitochondrial Membrane Potential Detection Kit (JC-1), and ATP assay kit were purchased from Dojindo Laboratories (Japan). All of the chemicals used in this study were of analytical grade and used without further purification.

### Synthesis of HSA-NO and preparation of PIH-NO

The albumin-based NO donor was obtained according to our previous reports 42. Briefly, HSA (800 mg) and 2-imminothiolane (16 mg) were co-dissolved in PBS (pH 7.8 and 0.5 mM diethylenetriamine pentaacetate) and reacted for 1 h at 37 °C. The resultant S-thiolated HSA was concentrated and reacted with isopentylnitrite (15 mM) for 3 h at 37 °C. The obtained HSA-NO was purified and quantified with BCA kit to obtain albumin concentration. The NO concentration was determined by Griess assay upon addition of mercury chloride (HgCl_2_).

PIH and PIH-NO were synthesized by ultrasonic methods. The IR780 dissolved in ethanol with different concentrations was added to HSA or HSA-NO solution (20 mg/mL) for 30 min. After that, FDC (400 μL) was gradually added under sonication (10 KHz, 200 W) in an ice bath for 4 min. The obtained PIH and PIH-NO were purified by ultrafiltration centrifuge tube (100 kDa) to remove IR780, HSA-NO and nitrite. The resulting nanoparticles were stored at 4°C for further use. As IR780 cannot be dissolved in PBS, the IR780 encapsulated in HSA (denoted as IH) was formed as control group based on our previous studies. Before use for any experiments, PIH-NO was bubbled O_2_ for 30 min to ensure sufficient chelation of O_2_ into FDC.

### Characterization of PIH-NO

The size distribution was determined by dynamic light scattering (DLS). The stabilities of PIH-NO in 37 °C and 4 °C were determined the size at 0, 6, 12, 24, 36, and 48 h. The size changes stored in PBS, culture medium, 5% HSA and plasma within 48 h at room temperature were also determined by DLS. Transmission electron microscopy (TEM) was applied to observe the morphology and structure of different formulations. The concentration of IR780 was determined by UV-vis spectrophotometer, and NO was quantified by a Griess assay. For the release of IR780 *in vitro*, 1 mL of PIH-NO (IR780, 153 μg/mL) was put into dialysis bag (MW=3000) and performed in 15 mL of PBS (pH=6.0 and 7.4, 0.01 M with 0.5% Tween80) and plasma. The release of IR780 at different time points was determined by the UV-vis spectrophotometer and calculated based on a standard line. The thermal effect of PIH-NO by US irradiation was evaluated by thermometer. 0.3 mL PBS and PIH-NO (IR780, 40 μg/mL) were irradiated by US (WED-110, Shenzhen, China) with 1.0 W/cm^2^ and determined by thermometer every 20s.

### Oxygen and NO release* in vitro*

The oxygen release behavior was determined by a portable dissolved oxygen meter (JBP, Shanghai). For US-triggered O_2_ release, PIH-NO (1 mL) with oxygen saturation was injected to deoxygenated water (5 mL with boiling under nitrogen atmosphere). The changes of oxygen concentration were recorded with time. After recording for 300 s, an external US treatment (1.0 MHz, 1 W/cm^2^) was applied for another 300 s, and the oxygen concentration was recorded for about 900 s in total. Deoxygenated water and oxygen saturated water were set as control groups.

To study the NO release profile with US treatment, PIH-NO (60 μM, 0.2 mL) was mixed with a Griess assay and irradiated by US (1.0 MHz, 1 W/cm^2^) for 4 min. The absorption was determined by UV-vis spectrophotometer every half minute. In addition, the NO release in different concentrations of PIH (20, 40, and 60 μM) was also determined by a Griess assay. We also determined the NO release of PIH-NO under various US powers (1.0 MHz, 0.5, 1.0, and 1.5 W/cm^2^). The PIH without NO donor group was set as the control.

### ^1^O_2_ and ONOO- generation

A specific fluorescent probe (SOSG) was applied to determine ^1^O_2_ generation of PIH-NO after US treated. The IH, PIH, and PIH-NO (200 μL, IR780 20 μg/mL, FDC 6%) and SOSG (10 μL, 50 μM) were added into black 96-well plates. After irradiated with US (1.0 MHz, 1 W/cm^2^), the fluorescence intensities of SOSG (Ex/Em = 495/525 nm) were recorded with various irradiation times after the generation of ^1^O_2_. The different concentrations of PIH, PIH-NO, and IH (IR780, 10, 20, and 60 μg/mL) were also determined by the SOSG method.

As for ONOO^-^ detection, the dihydrorhodamine 123 (DHR) was used as a fluorescence probe, which reacted with ONOO^-^ to produce rhodamine 123 with green fluorescence. PIH-NO and PIH (200 μL, IR780 20 μg/mL, FDC 6%) were mixed with DHR. The fluorescence spectra of rhodamine 123 from 500 nm to 600 nm were recorded after US treatment.

### Intracellular uptake of PIH-NO with or without HSA block

The 4T1 cells were incubated in 24-well plates (10^5^ per well) for 24 h. After that, HSA (10 mg/mL, 500 μL) was added to cells to block for 3h. Then, the culture medium of 4T1 cells with or without HSA was removed and incubated with PIH-NO for another 3 h. Finally, the cells were incubated with DAPI and washed with PBS to observe the fluorescence of IR780 (Ex/Em:633/700-800 nm) by confocal microscope (OLYMPUS, FV1000).

### Mitochondria-targeting and disruption of PIH-NO

The 4T1 cells were incubated in 24-well plates (10^5^ per well) for 24 h. The PIH-NO (IR780, 8 μg/mL) were then incubated with 4T1 cells for 3 h. The cells were then washed with PBS and stained with Mitotracker Green (100 nM, Ex/Em = 490/516 nm) for 30 min. Finally, the cells were incubated with DAPI and observed by confocal microscope (OLYMPUS, FV1000). The lysosome staining was similar as described above and the tumor cells were incubated with Lyso-tracker Green (50 nM, Ex/Em =504/511 nm). In order to confirm the mitochondria-targeting property of PIH-NO, FITC (50 μL, 10 mg/mL in DMSO) was added into PIH-NO or PH-NO solutions and carried out overnight at 4 ℃ based on previous studies. Then, FITC labeled PIH-NO nanoparticles were obtained by dialyzing against PBS. The other methods were similar as described above. The fluorescence of FITC (Ex/Em: 490/520 nm), Mitotracker Red (Ex/Em: 561/600 nm) and IR780 (Ex/Em: 635/700-800 nm) were imaged by confocal microscope (OLYMPUS, FV1000).

MitoPeDPP (Ex/Em:470/525 nm) is a fluorescent dye to monitor mitochondria; it emits a strong green fluorescence after being oxidized by lipophilic peroxides. This label was applied to determine the oxidization of mitochondria membranes. The 4T1 cells were incubated in 96-well plates and incubated for 24 h. The cells were treated with IH, PIH, and PIH-NO (8 μg/mL, FDC 3%) and then treated with US for 1 min. The cells were then washed with PBS and incubated with MitoPeDPP working solution for 15 min according to the protocol. The fluorescence images of 4T1 cells were captured by fluorescence microscope (Nikon, Japan).

The mitochondrial membrane potential detection kit (JC-1) was also applied to determine the disruption effect of PIH-NO in mitochondria. Here, 4T1 cells were treated with IH, PIH, or PIH-NO (10 μg/mL) for 2 h. The JC-1 work solution was then added and stained for 40 min. After that, cells were washed and exposed to US (1.0 MHz, 1 W/cm^2^) for 1 min. The fluorescence images of JC-1 were captured by a confocal microscope (OLYMPUS, FV1000).

### Intracellular ROS and NO generation

To avoid the effect of SDT, PH and PH-NO without IR780 were also applied to evaluate the reversal of the hypoxic environment. PH and PH-NO (FDC 3%, NO 50 μM) were mixed with hypoxia detection reagent (1 μM) and incubated with 4T1 cells for 3 h. The upper medium was covered with liquid paraffin to produce hypoxia (4%-5% oxygen). The cells were then washed and stained with DAPI for fluorescence imaging. After being treated with IH, PIH, and PIH-NO (IR780 20 μg/mL, FDC 3%, NO 10 μM), 4T1 cells were stained with H_2_DCFDA (ROS indicator, 20 μM) or DAF-FM DA (NO indicator, 5 μM), respectively. After US treatment (1.0 MHz, 1 W/cm^2^) for 1 min, the cells were washed and incubated with DAPI for 15 min. Finally, the fluorescence images were captured by fluorescence microscope (Nikon, Japan). The fluorescence of DAF-FM DA was also evaluated by fluorescence microplate based on above experiments. The ROS generation was also evaluated by a flow cytometer (ACEA).

### *In vitro* cytotoxicity

To investigate the therapeutic efficacy of SDT* in vitro*, 4T1 cells (10^4^ per well) were seed in 96-well plates, cultured overnight, and incubated with various concentrations of IH, PIH, and PIH-NO for 3 h. After washing with PBS, 4T1 cells were treated with US (1.0 MHz, 1 W/cm^2^) for 1 min or without any treatment. After incubation for another 4 h, the culture medium was removed, and CCK-8 was added to determine the cell viability. Calcein AM/PI staining was also applied to evaluate the SDT efficacy of PIH-NO. The procedure was similar as described above. After being washed with PBS, a mixture of PI (8 μM) and Calcein AM (2 μM) were added for 15 min. The fluorescence images were obtained by fluorescence microscope (Nikon, Japan). In addition, Annexin V-FITC/PI double staining was also used to detect apoptosis by flow cytometry (ACEA).

### CRT expression, ATP, and HMGB-1 release

CRT expression as well as extracellular release of ATP and HMGB-1 are three main ICD markers. To evaluate the ICD of PIH-NO, CRT exposure in the surface of 4T1 tumor cells was determined by immunofluorescence staining. After incubation with IH, PIH, or PIH-NO (10 μg/mL) for 3 h, the culture medium was carefully moved to a tube for ATP and HMGB-1 determination. The extracellular ATP was determined by bioluminescence assays with ATP assay kit (Beyotime, Nanjing, China) based on the standard protocols. The light emission was measured with Multi-mode board reader. The HMGB-1 were also determined by a HMGB-1 elisa kits (Tongwei, Shanghai, China) according to the manufacture's protocol. Then, Otherwise, the 4T1 cells were washed with PBS and irradiated with US (1.0 MHz, 1 W/cm^2^) for 1 min or without any treatment. The cells were then incubated with CRT primary and secondary antibodies according to standard protocols. The fluorescence was observed by fluorescence microscope (Nikon, Japan). The mean fluorescence intensity was calculated based on Image J software.

### Animal model

BALB/C male mice were obtained from Yangzhou University Medical Center (Yangzhou, China). All of the mice received care following the guidelines of the Care and Use of Laboratory Animals, and their use followed the terms of the Institutional Animal Care regulations and Use Committee of Nanjing University. All of the animal experiments were approved by the Administration Committee of Experimental Animals in Jiangsu Province and the Ethics Committee of Nanjing University. The subcutaneous 4T1 bearing mice were established by injection of 4T1 cells (10^5^ per mouse) in the lower flanks.

### Biodistribution and pharmacokinetics of PIH-NO *in vivo*

To avoid autofluorescence interference, subcutaneous 4T1 tumor-bearing mice were established by injection of 4T1 cells (10^5^ per mouse) in the flank. After the tumors reached 200 mm^3^, PIH or PIH-NO (200 μL, IR780, 78 μg/mL) were intravenously injected. The fluorescent imaging was performed at predetermined time (2, 4, 6, 12, 24, and 36 h). At the end of experiment, tumors and major organs were obtained for fluorescence imaging. The semi-quantitative biodistribution analysis was calculated via fluorescence. In order to further confirm the influence of US irradiation in tumor accumulation, 4T1 tumor-bearing mice were intravenously injected with PH and PH-NO (50 μM, 200 μL). After 24 h post-injection of PH-NO and irradiated with US treatment (1.0 MHz, 1 W/cm^2^, 5 min), Evans blue (5 mg/mL, 200 μL) was intravenously injected into 4T1 tumor bearing mice. Then, the tumors were collected to weigh and perform frozen slices. The fluorescence images of Evans blue were obtained by fluorescence microscope. The concentrations of Evans blue in tumor were determined by UV-vis spectrophotometer at 620nm.

The blood perfusion was determined by doppler flowmeter (Moor VMS) at different time points (0, 6, 12, and 24 h). The immunofluorescence staining of HIF-1α was also performed at 24 h post injection of treated groups. The anti-HIF-1α primary and FITC-secondary antibody were incubated with slices according to recommended procedures. The pharmacokinetics of PIH-NO and PIH were studied in BALB/C male mice. The absorption of IR780 is 780nm, which can avoid the interference of blood. So, after intravenous injection of PIH-NO and PIH (IR780, 120 μg/mL, 200 μL), the blood was collected at different time points and detected by NanoDrop 2000. The concentration of IR780 was calculated based on standard line.

### Anti-tumor and metastasis *in vivo*

When the tumor reached approximately 100 mm^3^, 4T1 bearing mice were randomly divided into six groups: saline, saline+US, IH+US, PIH+US, PIH-NO, and PIH-NO+US. Mice were intravenously injected with the various treatments (200 μL, IR780 200 μg/ml, NO 140 μM, and PFC 6%). The ultrasound (1.0 MHz, 1 W/cm^2^) by ultrasonic therapeutic apparatus (WED-110, Shenzhen, China) was applied at the tumor site for 5 min 24 h post-injection. After various treatments, the lengths and widths of tumors were measured for a period of 14 days and calculated according to the formula: volume = width^2^ × (length/2). At the end of treatments, the mice were anesthetized to remove tumors, and the wound site was closed with sutures. The tumors were weighed to evaluate the therapeutic efficacy. The mice were then maintained for another three weeks to evaluate the lung metastasis. The lungs were collected for H&E staining. The tumors were obtained to stain with H&E and TUNEL according to standard protocols on day 3 after various treatments.

### Immunosuppression of TME reprogramming

To evaluate the immunosuppression and TME reprogramming, the tumors were collected at day 3 after various treatments. Here, M1, M2, and MDSC in tumors after various treatments were evaluated by flow cytometry according to standard protocols. For analysis of macrophages, the acquired and suspended cells were stained with anti-CD11b-FITC, anti-F4/80-PE/Cy7, anti-CD86-PE, and anti-CD206-APC; cells were monitored by FCM to analyze M1 or M2 macrophages. MDSCs were stained by antibodies for CD11b-FITC and Gr-1-APC (all from BioLegend, San Diego, CA, USA).

### Antitumor immune response

Induction of ICD was evaluated by CRT exposure on dying tumor cells and HMGB-1 release. The tumor-draining lymph nodes (TDLNs) and tumors were collected at day 3 after different treatments. CRT or HMGB-1 primary and fluorescence labeled secondary antibody were incubated with frozen tumor section. The fluorescence images were captured by a fluorescence microscope (Nikon, Japan). The suspended cells from TDLN were stained with anti-CD11c-FITC, anti-CD86-PE, and anti-CD80-APC (all from BioLegend, San Diego, CA, USA), and then analyzed by flow cytometry for DC maturation.

To investigate the anti-tumor response, tumors were collected on day 7 after various treatments. They were dissociated to produce a single-cell suspension. Cells were stained with anti-CD3-APC and anti-CD8-PE for cytotoxic T lymphocytes (CD3^+^CD8^+^) and analyzed by FCM. The immunofluorescence staining of CD8 and IFN-γ were observed in tumor slices. In addition, the levels of TNF-α in the tumor were measured by an ELISA kit according to the standard protocols.

### Biosafety *in vivo*

Six female ICR mice were randomly assigned to two groups (saline and PIH-NO) for biosafety evaluation. After intravenous injection with saline and PIH-NO (IR780 2 mg/kg), the blood was collected to analyze biochemical markers (ALT, AST, ALP, BUN, and CREA). Major organs were harvested, and H&E staining was performed.

### Statistical analysis

The comparisons between different groups were analyzed by two-sided student's t-test. Significance was expressed as *p < 0.05 and **p < 0.01.

## Results and Discussion

### Preparation and characteristics of PIH-NO

**Figure 2:**
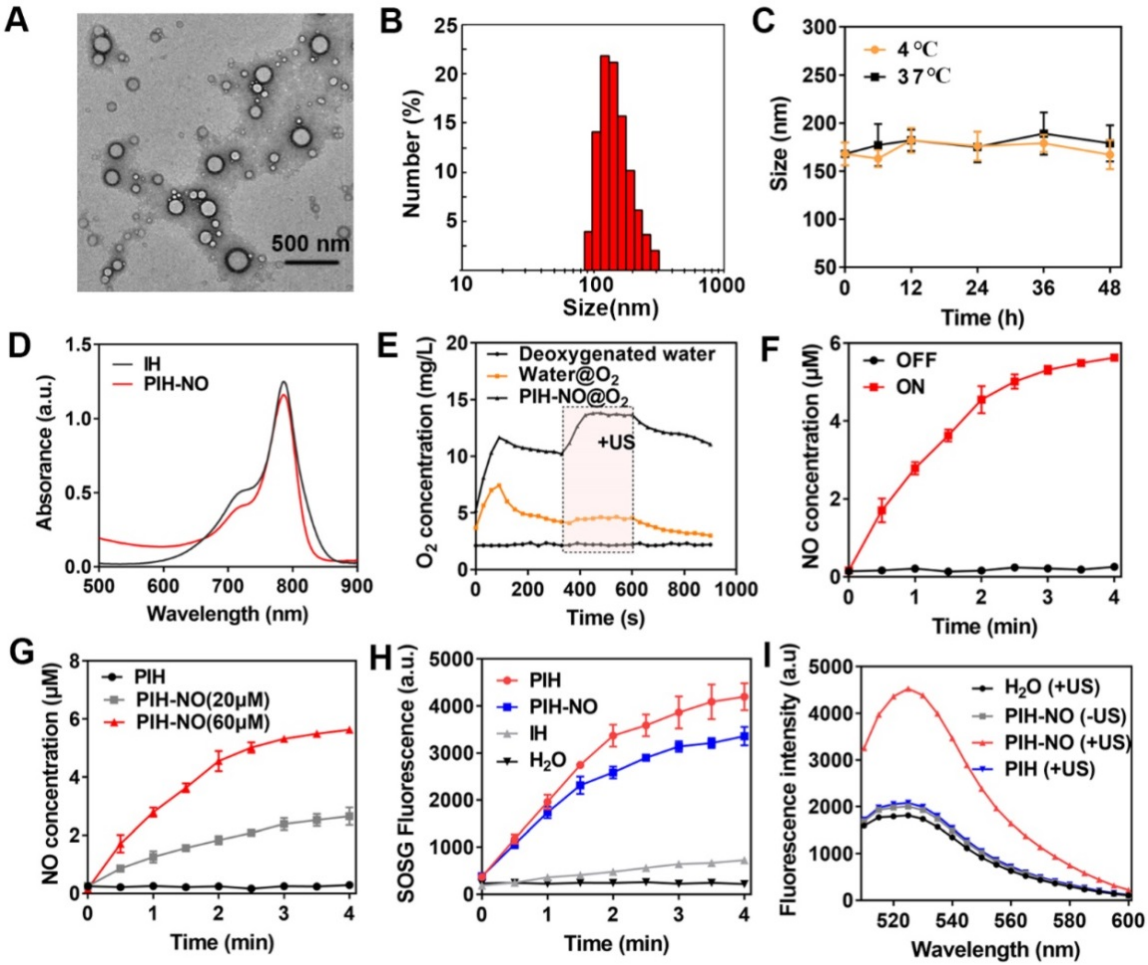
Characteristic of PIH-NO. (A) TEM images and (B) size distribution of PIH-NO. (C) The stability of PIH-NO within 48 h when stored at 4 °C and 37 °C. (D) UV-vis absorption spectra of IH and PIN-NO. (E) Time-dependent changes of dissolved oxygen concentrations in deoxygenated pure water after addition of water@O_2_ and PIH-NO@O_2_ with US treatment in the indicated period (6 min to 10 min). (F) The release of NO in PIH-NO (NO 60 μM) with or without US treatment. (G) The release of NO with different concentrations of PIH-NO (0, 20, and 60 μM). (H) The generation of singlet oxygen under US treatment determined by SOSG fluorescence. (I) Fluorescence spectrum of ONOO^-^ in different groups evaluated by dihydrorhodamine 123 (DHR). Unless otherwise specified, US treatment is under 1.0 MHz and 1.0 W/cm^2^.

HSA-NO was synthesized according to previous studies. HSA was modified with 2-iminothiolane to introduce sulfhydryl groups (-SH) and then reacted with isopentyl nitrite to form S-NO, which showed a characteristic peak at 340 nm (Figure S1). The concentration of NO in HSA-NO was 5.1 mol NO/ mol HSA as determined by the Griess assay upon the addition of HgCl_2_. Owing to the hydrophobic characteristics of IR780, it can strongly bind to HSA-NO to form the complex IR780-HSA-NO. After the addition of FDC (6% v/v), HSA-NO was used as a stabilizer to form the PIH-NO nanoemulsion. The encapsulation of FDC was approximately 100% in PIH-NO without any FDC leakage. As shown in Figure 2A, the transmission electron microscopy (TEM) image showed that PIH-NO was well-dispersed with a spherical structure. The average hydrodynamic diameter was around 168 nm (polydispersity index, PDI=0.29), as determined by dynamic light scattering (Figure 2B). In addition, the other formulations of IH and PIH showed the same particle sizes as that of PIH-NO (Figure S2). More importantly, there was no obvious changes in the particle size after storage at 4 or 37°C within 2 days, suggesting that PIH-NO possessed good stability (Figure 2C). Moreover, the changes in size of PIH-NO were evaluated in PBS, culture medium, 5% HSA and plasma at room temperature. As shown in Figure S3, after the addition of PIH-NO to 5% HSA and plasma, the original size of PIH-NO increased, by approximately 40 nm due to the absorption of proteins. However, there were no significant changes within the storage of 48 h, which further indicated that PIH-NO possessed high stability.

The characteristic peak of IR780 was observed in the PIH-NO spectrum, which indicated the successful encapsulation of IR780 (Figure 2D). In addition, the release of IR780 in PBS (pH=7.4 and 6.0) and plasma was evaluated by UV-vis spectroscopy. As shown in Figure S4, only approximately 2.9% of the IR780 leaked out from the PIH-NO nanoparticles in PBS (pH=7.4) and plasma within 24 h. Additionally, in the acidic environment, only 13.8% of the IR780 was released from PIH-NO nanoparticles. This may be due to the high hydrophobicity of IR780 45. These results indicated that PIH-NO showed high stability and was suitable for further study. The concentration of NO in PIH-NO was determined by the Griess assay, and the encapsulation efficiency was around 48.3%. These results indicated the successful preparation of PIH-NO.

### US-responsive release of O_2_ and NO

The release of O_2_ with US treatment was determined by a portable dissolved oxygen meter. The amount of O_2_ dissolved in PIH-NO rapidly increased to 12.8 mg/L within 120 s after addition the addition of O_2_-saturated PIH-NO and water into deoxygenated water (under nitrogen atmosphere). This value was higher than that of water@oxygen injection (7.6 mg/L) due to the high O_2_-carrying capacity of FDC (Figure 2E). PIH-NO also showed a slower decrease than that of water, thus maintaining the O_2_ in FDC without release. For SDT cancer treatment, the energy activated by sonosensitizers is transferred to O_2_ for ^1^O_2_ generation. Thus, it is necessary to release oxygen from FDC to enhance SDT. After being triggered by low-power and low-frequency US (1.0 MHz, 1 W/cm^2^), a burst-like release of oxygen was observed, as indicated by the dissolved O_2_ concentration (14.8 mg/L). This burst lasted longer during the US treatment (240 s). The results indicated that PIH-NO could load more O_2_ and respond to US-mediated release for SDT enhancement.

US can induce the decomposition of -SNO to NO. As shown in Figure 2F, the release of NO from PIH-NO increased with US treatment time and was highly dependent on US irradiation. In addition, NO release was also dependent on US power (Figure S5) and the concentration of PIH-NO (Figure 2G). This result is due to the transient and extremely high temperature and pressure generated by ultrasonic cavitation and sonoluminescence. The thermal effects of PIH-NO with US treatment were also evaluated with a thermometer. As shown in Figure S6, after US treatment, the temperature of PIH-NO and PBS increased with irradiation time. The temperature of the PIH-NO solution (IR780, 40 μg/mL) increased about 7 ℃ within 4 min, which was higher than that of the PBS group (approximately 3 ℃). These results indicated that IR780 could be activated by US to transfer energy at a slightly increased temperature.

### ^1^O_2_ and ONOO- generation of PIH-NO

During US-triggered O_2_ release, the enhancement in SDT was evaluated by singlet oxygen sensor green (SOSG) to determine ^1^O_2_ generation. As shown in Figure 2H and S7, after US treatment, PIH-NO and PIH showed a significant increase in ^1^O_2_ generation compared with that of IH due to the high oxygen loading of FDC. The final ^1^O_2_ generation of PIH-NO occurred slightly slower than that of PIH. This may be due to the consumption of ^1^O_2_, which destroys -SNO to release NO or oxidizes NO to form the strong oxidant peroxynitrite (ONOO-). The ability of ONOO- generation was examined by DHR, which is a specific ONOO- analytical reagent that emits strong green fluorescence at 526 nm. As shown in Figure 2I, the fluorescence of DHR in the PIH-NO group increased significantly after US treatment, while the PIH-NO without US and PIH with US groups showed no DHR fluorescence signal. This result confirmed that a large amount of ONOO- was formed by the reaction between NO and ROS after US irradiation. These results indicated that our formed PIH-NO could respond to US for oxygen and NO release to enhance SDT and generate strong ONOO- for cancer treatment.

### Cellular uptake and mitochondrial-targeting property of PIH-NO

**Figure 3:**
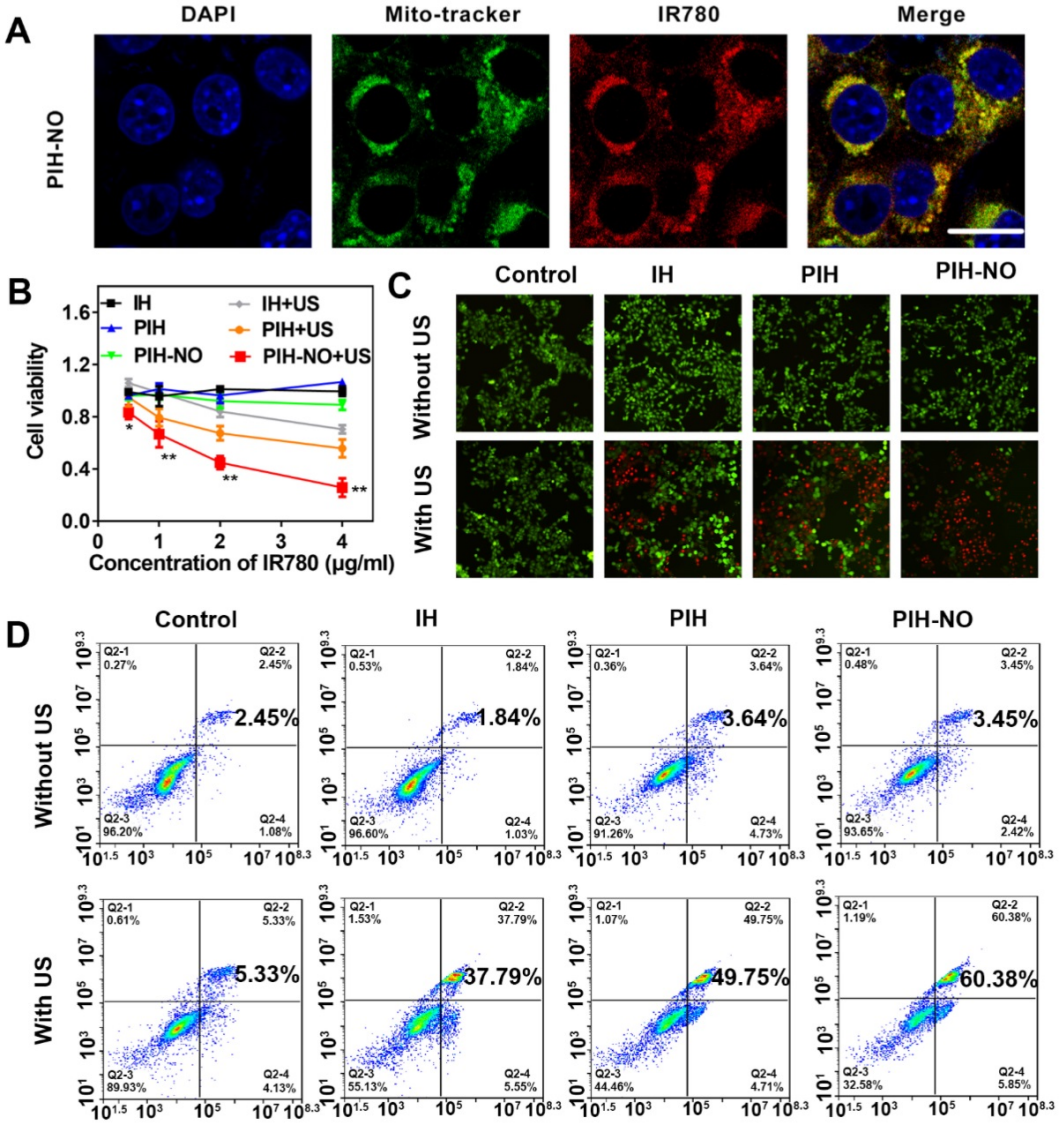
The cell viability of mitochondria-targeted PIH-NO *in vitro*. (A) Mitochondrial location of PIH-NO monitored by Mito-tracker fluorescence. Blue, green, and red represent DAPI, Mito-tracker Green, and IR780 fluorescence, respectively. The scale bar is 20 μm. (B) Cell viabilities of 4T1 cells after incubation with different groups at various doses. *p <0.05, **p <0.01 (PIH-NO with US vs. other treatments). (C) Calcein-AM (green)/PI (red) staining and (D) flow cytometry apoptosis of 4T1 cells after various treatments. The scale bar is 100 μm. Data are expressed as means ± SD (n = 3).

Based on previous studies, HSA nanocarriers show high affinity for gp60, which is highly expressed in tumor cells 46. Therefore, HSA was used to block gp60 to evaluate cellular uptake. The cellular uptake was performed in 4T1 tumor cells based on the fluorescence intensity of IR780. As shown in Figure S8, bright red fluorescence was observed after incubation with PIH-NO for 3 h. When blocked with HSA, the red fluorescence of IR780 decreased significantly, which indicated that PIH-NO may have increased cellular uptake. In addition, as shown in Figure S9, there was no overlap between the red fluorescence of IR780 and the green fluorescence of lysosomes. This result indicated that after entering tumor cells, PIH-NO could escape from lysosomes, which is similar as previous study. More importantly, IR780 can preferentially accumulate and be retained in mitochondria 47. Next, 4T1 cells were stained with MitoTracker Green to verify whether PIH-NO could target the mitochondria of cancer cells after incubation with PIH-NO. As shown in Figure 3A, the red fluorescence of IR780 showed a gradual overlap with the green fluorescence of Mito Tracker Green, which indicated intracellular colocalization of PIH-NO with mitochondria. This result suggested the high mitochondria-preferential accumulation of PIH-NO due to the encapsulation of IR780.

To further confirm the mitochondrial-targeting of PIH-NO, FITC was conjugated to HSA to form FITC-labeled PIH-NO and PH-NO. As shown in Figure S10, without IR780, the green fluorescence of FITC did not fully overlap with the purple fluorescence of MitoTracker (PC=0.15), which indicated that PH-NO entered into tumor cells and was widely distributed in the cells. After incubation with the FITC-labeled PIH-NO, the green fluorescence of FITC and red fluorescence of IR780 highly overlapped with mitochondria (PC=0.76). All of these results indicated that IR780 could induce PIH-NO to accumulate in mitochondria.

### *In vitro* cytotoxicity

Enhanced SDT was evaluated *in vitro* in 4T1 tumor cells by CCK-8 assays. As shown in Figure 3B, without US treatment, IH, PIH, and PIH-NO all showed negligible cytotoxicity. Upon US irradiation, SDT efficacy was found to be PIH-NO dose-dependent. At the same concentration of IR780 (4 μg/mL), PIH-NO showed approximately 70% inhibition of tumor cells, which is higher than that of PIH (50%) and IH (30%) with US treatment. Furthermore, calcein-AM (green fluorescence) and propidium iodide (PI, red fluorescence) staining were applied to visualize live and dead cells, respectively. Treatment with PIH-NO plus US irradiation showed stronger red fluorescence than the other groups, revealing increased cell death through enhanced SDT and NO gas therapy (Figure 3C). After various treatments, the 4T1 cells were stained with annexin V-FITC and PI and analyzed by flow cytometry to further corroborate the therapeutic efficacy of PIH-NO. As demonstrated in Figure 3D, the apoptosis/necrosis rate of cells treated with PIH-NO was 60.38%, which was approximately 2-fold higher than that of IH with US (37.79%). These collective results indicated that US-triggered NO and oxygen release could enhance SDT therapeutic efficacy.

### Reversion of hypoxia *in vitro*

**Figure 4:**
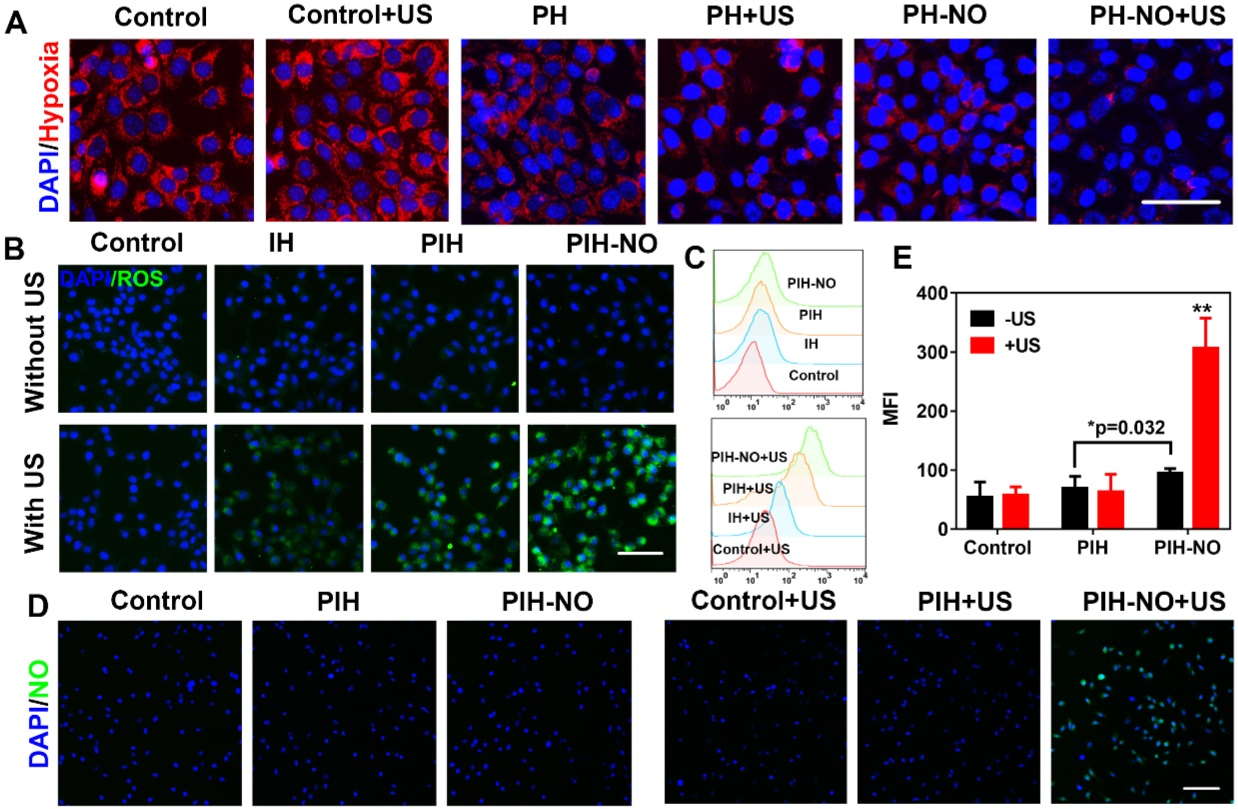
Intracellular ROS and NO generation. (A) Representative fluorescence images of hypoxia (red) in 4T1 tumor cells after different treatments. (B) ROS generation (DCFH-DA, green) and (C) Flow cytometry quantitative analysis of ROS generation in treated groups with or without US. The scale bar is 100 μm. (D) Representative fluorescence images and (E) semiquantitative analysis of NO release stained with DAF-FM (green) in 4T1 tumor cells after various treatments. The scale bar is 200 μm. *p <0.05 (PIH-NO without US vs. PIH without US), **p <0.01 (PIH-NO with US vs. other treatments). Data are expressed as means ± SD (n = 3).

Owing to the release of O_2_ and NO from PIH-NO triggered by US, we investigated the intracellular oxygen level with a hypoxia detection kit. To prevent the consumption of oxygen during the SDT process, 4T1 cells were treated with PH and PH-NO without IR780 under hypoxic conditions for intracellular oxygen determination (cells were covered with liquid paraffin). The control groups showed bright red fluorescence while PH-NO treated with US showed slight red fluorescence, indicating that PH-NO could reverse hypoxia through the release of O_2_ and NO (Figure 4A). We also found that PH-NO showed less red fluorescence than PH group due to the NO released by GSH in tumor cells (Figure S11). It has been reported that NO can be triggered by GSH to block respiration and suppress oxygen consumption. These results indicated that PIH-NO responded to US to release oxygen and NO and reverse hypoxia in the TME.

### Intracellular ROS and NO generation with US treatment

Intracellular ROS generation is recognized as the main mechanism of SDT for cancer treatment. Therefore, the increase in intracellular ROS generation was evaluated by H_2_DCFDA fluorescence imaging and flow cytometry. As shown in Figure 4B, 4T1 cells treated with PIH-NO plus US irradiation showed strong green fluorescence, which was more intense than that in the IH and PIH plus US irradiation groups. The four treatment groups without US irradiation showed negligible fluorescence signals. The trends observed after DCF staining were further evaluated by quantitative flow cytometry analysis (Figure 4C). These results indicated that upon US treatment, the ROS generation of SDT was significantly increased due to oxygen and NO release.

The NO indicator (DAF-FM DA) and ONOO- indicator (DHR) were incubated with 4T1 cells to verify NO release from PIH-NO and ONOO- generation, respectively. With US treatment, PIH-NO showed strong green fluorescence, that was more intense than that in the other groups (Figure 4D). The fluorescence signal in the PIH-NO without US irradiation was minor due to the presence of GSH in TME that triggered NO release (Figure 4E). These results were confirmed by determining the fluorescence of NO with microplates, which showed similar results as that of fluorescence images (Figure S12). Furthermore, the fluorescence images of ONOO- displayed the same trend as that of NO release (Figure S13). Cells treated with PIH without NO did not exhibit any fluorescence regardless of US irradiation (Figure S14). All of these results indicated that PIH-NO could be triggered by US to release oxygen and NO for SDT enhancement.

### Destruction of mitochondria by PIH-NO

**Figure 5:**
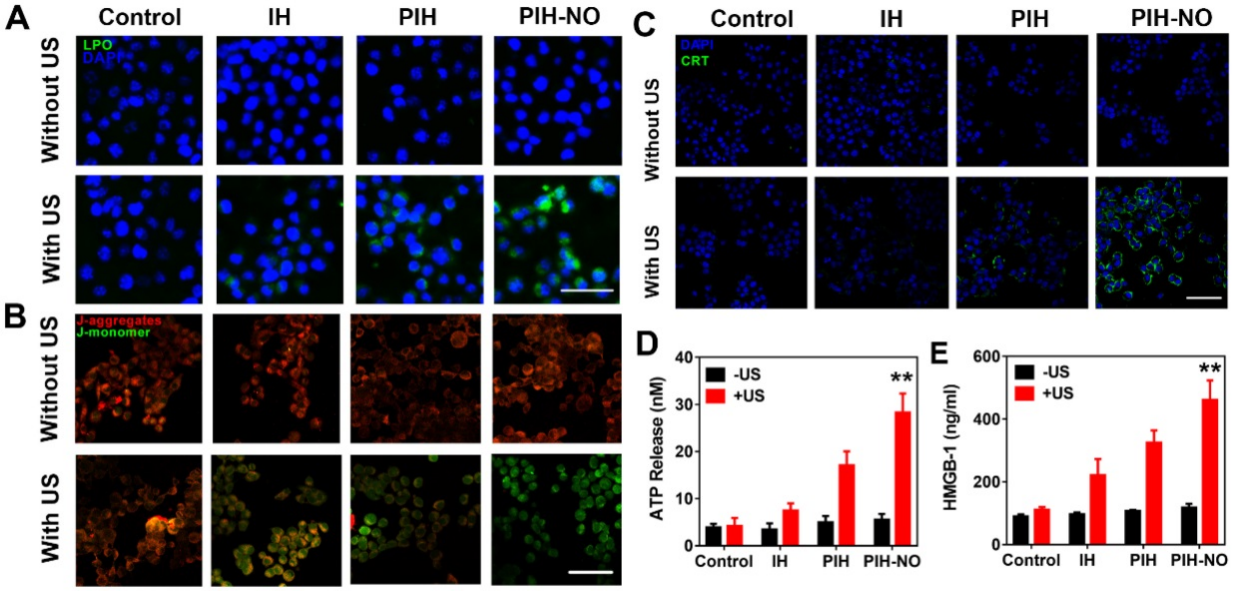
Mitochondria destruction and ICD induced by PIH-NO. (A) Fluorescence images of lipid peroxide in 4T1 cells with different treatments detected by Liperfluo (green fluorescence) for the lipid peroxide-specific oxidation. The scale bar is 100 μm. (B) Mitochondrial membrane potential stained with JC-1 after various treatments. The scale bar is 100 μm. (C) CRT exposure on the surface of 4T1 cells. The scale bar is 100 μm. (D) Extracellular ATP and (E) HMGB-1 release from 4T1 tumor cells after various treatments. **p <0.01 (PIH-NO with US vs. other treatments). Data are expressed as means ± SD (n = 3).

As described above, PIH-NO could preferentially accumulate in the mitochondria and produce ROS and NO for enhanced SDT. Therefore, we next evaluated the destruction of mitochondria by PIH-NO. MitoPeDPP can accumulate in the mitochondria and emit strong green fluorescence after being oxidized by lipophilic peroxides. 4T1 cells treated with PIH-NO plus US irradiation showed bright green fluorescence that was higher than in the other groups (Figure 5A and S15). No obvious fluorescence signal was observed in the treatments without US irradiation. These results indicated that the enhanced SDT and NO gas release oxidized the mitochondrial membrane to induce cell apoptosis.

The fluorescent probe JC-1 was then applied to further monitor the mitochondrial membrane potential: It emits green fluorescence to indicate mitochondrial damage and red fluorescence in the presence of normal mitochondria. The trends observed with JC-1 were similar to the mitochondrial peroxide results indicated by MitoPeDPP. Obvious green fluorescence was observed in the group of PIH-NO plus US irradiation, while the other PIH and IH with US groups showed slight green fluorescence (Figure 5B and S16). The collective data indicated that PIH-NO could accumulate in the mitochondria and increase ROS and NO generation under US to oxidize the mitochondrial membrane for cancer treatment.

### ICD amplification* in vitro*

Intracellular ROS by SDT could significantly induce immunotherapy-related ICD to activate an antitumor immune response 48. The common markers used to confirm ICD are calreticulin (CRT) exposure on dying tumor cells, HMGB-1, and ATP release. These markers could stimulate antigen presentation and induce immune response. After 4T1 cells were treated with various treatments, CRT expression was determined with CLSM by staining with CRT antibodies. As shown in Figure 5C, CRT exposure on the cell membrane was clearly observed after PIH-NO plus US irradiation. Additionally, PIH with US treatment showed slight green fluorescence due to the enhanced SDT with FDC. The extracellular contents of HMGB-1 and ATP were determined and showed trends similar to that of CRT expression (Figure 5D and E). These results indicated that PIH-NO with enhanced SDT and NO release could amplify ICD by CRT expression and HMGB-1 release to potentially provoke an immune response.

### Biodistribution of and blood perfusion

The pharmacokinetics of PIH-NO and PIH were evaluated *in vivo* by intravenous injection of 2 mg/kg IR780. IR780 absorbs in the NIR region at 780 nm, avoiding the interference of blood 49. At the indicated time points, blood was collected and centrifuged to determine the concentration of IR780. As shown in Figure S17 and Table S1, after intravenous injection of PIH-NO and PIH, the concentration of IR780 decreased with circulation time, but there was no significant difference between these two groups. This may be due to the similar structures and stabilities of these two nanoparticles under physiological conditions.

**Figure 6:**
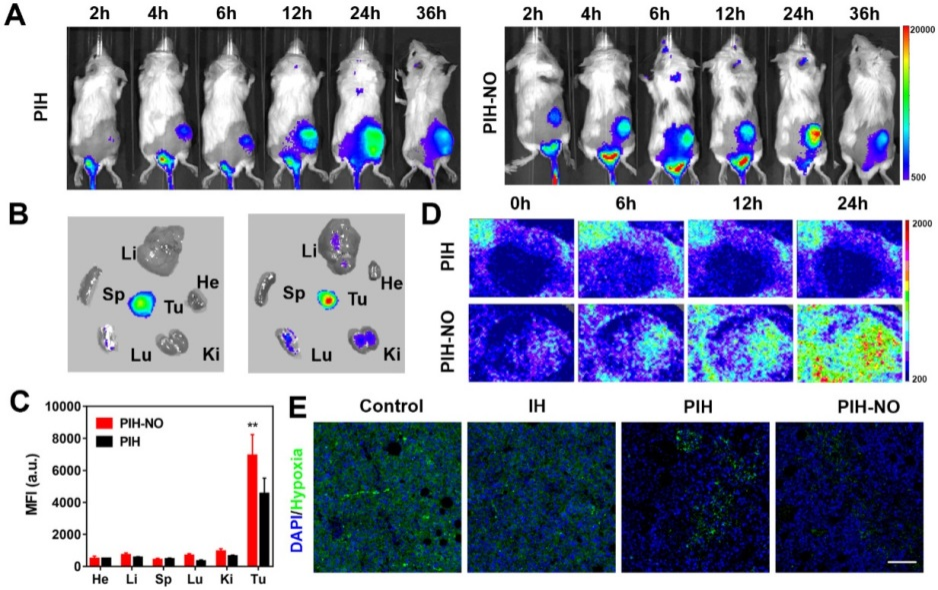
Biodistribution of PIH-NO *in vivo*. (A) Real-time fluorescence images of 4T1 tumor-bearing mice after intravenous injection of PIH and PIH-NO (IR780, 78 μg/mL). (B) *Ex vivo* fluorescence images and (C) the corresponding relative mean fluorescence intensity of major organs and tumor at 36 h after PIH and PIH-NO treatments (**p <0.01, PIH-NO vs. PIH). (D) The blood perfusion detected by a Doppler flowmeter in the tumor after various treatments. (E) Immunofluorescence images of HIF-1α standing of tumor slices 24 h post injection of different formulations. The scale bar is 200 μm. Data are expressed as means ± SD (n = 3).

NO plays an important role in vascular smooth muscle. It enhances vascular permeability and blood flow to enhance nanoparticles accumulation in tumors 50, 51. Fluorescence imaging was performed to determine the distribution of PIH-NO in 4T1 tumor-bearing mice after intravenous injection. As shown in Figure 6A, the fluorescence signal of IR780 increased with time and peaked at 24 h post injection. Compared with PIH, the accumulation of PIH-NO was nearly 1.4-fold higher at 24 h post-injection (Figure S18), which indicated that the NO triggered by GSH in the TME could enhance nanoparticle accumulation. After intravenous injection at 36 h, the tumor and major organs were collected, and fluorescence signal from IR780 was acquired. As shown in Figure 6B and 6C, the accumulation of PIH-NO in the tumors was higher than that in PIH. All of these results indicated that the NO released from PIH-NO could increase tumor accumulation by the enhanced permeability and retention (EPR) effect.

To avoid the influence of IR780, the EPR enhancement of US-treated NO was evaluated by Evans blue after intravenous injection of PH-NO and PH. Evans blue dye can bind to albumin and extravasate into tumors *in vivo* after intravenous injection, which is considered a marker to evaluate the EPR effect 52. As shown in Figure S19, without US treatment, PH-NO showed a higher intensity of red fluorescence than PH, which indicated that GSH-induced NO production enhanced the EPR effect, similar to the results of fluorescence image. After US irradiation, the red fluorescence in the PH-NO group was increased substantially compared with all other treatments. In addition, the concentration of Evans blue in the PIH-NO with US was about 2.4-fold higher than PIH-NO without US (Figure S20). These results indicated that US-induced NO release could increase the EPR effect to enhance tumor accumulation for cancer treatment.

Blood perfusion was examined in the tumors by Doppler flowmeter after various treatments. As shown in Figure 6D and S21, blood perfusion in the PIH-NO group increased with time and was higher than that in the PIH group. These findings confirmed that NO could increase blood perfusion to increase nanoparticle accumulation. Immunofluorescence staining of HIF-1α was performed to evaluate the changes in tumor hypoxia 24 h after intravenous injection (Figure 6E). The group treated with PIH-NO showed less intense green fluorescence than the other groups, which indicated that FDC with oxygen and NO release could retard hypoxia* in vivo*.

### Tumor and metastasis inhibition* in vivo*

**Figure 7:**
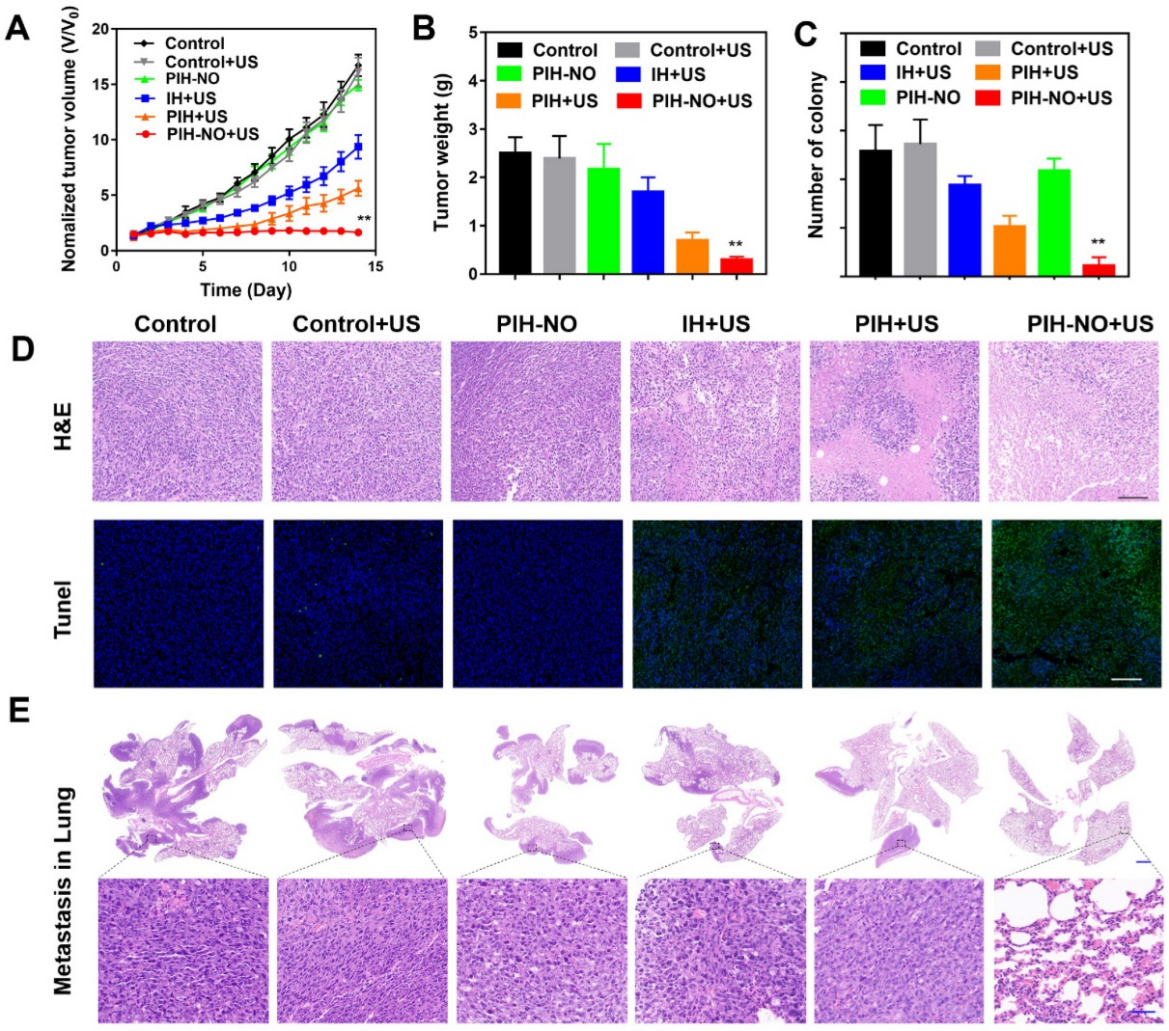
Inhibition of tumor and metastasis of PIH-NO *in vivo*. (A) Tumor growth curves and (B) average tumor weight after various treatments. (C) The number of lung metastasis. (D) H&E and TUNEL staining of tumor slices after various treatments on the 14th day. The scale bar is 200 μm. (E) H&E staining of lung metastatic nodules. The scale bar is 100 μm. *p <0.05, **p <0.01 (PIH-NO with US vs. other treatments). Data are expressed as means ± SD (n = 6).

Encouraged by the increased tumor accumulation and hypoxia relief of PIH-NO, the therapeutic efficacy of SDT and NO gas therapy was next evaluated in 4T1 tumor-bearing mice. When the tumor volumes reached approximately 100 mm^3^, the mice were divided randomly into the following six groups (n = 6): saline, saline+US, PIH-NO, IH+US, PIH+US, and PIH-NO+US (US treatment: 1.0 MHz, 1 W/cm^2^, 5 min). As shown in Figure 7A, PIH-NO with US irradiation combined treatment completely eradicated the tumor. Additionally, PIH and IH with US treatment showed a significant inhibition of tumors versus the control group. Moreover, the tumor weights were consistent with relative tumor volume results (Figure 7B and S22). The inhibition rate of PIH-NO with US treatment was approximately 92%, which was higher than that of IH with US treatment (27%) and PIH with US treatment (63%). These results indicated that the enhanced SDT and NO therapy could significantly suppress tumor growth due to higher accumulation and hypoxia relief.

To further evaluate the therapeutic efficacy of PIH-NO, H&E and TUNEL staining assays were performed three days after the various treatments. As shown in Figure 7D, destructive cell necrosis was observed in the group treated with PIH-NO plus US irradiation. There were only a few apoptotic cells observed in IH and PIH with US treatments. All of these values were higher than those of the control and PIH-NO without US treatments, which showed no obvious cell damage. The TUNEL staining results were consistent with the H&E staining results. Strong green fluorescence was observed in the group of PIH-NO with US irradiation. In addition, the number of pulmonary metastatic nodules was quantified to further evaluate the antimetastatic efficacy of PIH-NO, and the number of colonies significantly decreased (Figure 7C). Few lung metastases were observed by H&E staining of the lung tissue slices of mice treated with PIH plus US irradiation (Figure 7E). These collective results indicated that the enhanced combination of SDT and NO from PIH-NO with US could significantly inhibit tumor growth and lung metastasis.

The biosafety of PIH-NO was evaluated by body changes, H&E staining of the major organs, and blood biochemical markers. As shown in Figure S23 and S24, there were no significant changes in the weights of the mice and no damage to the major organs was observed after various treatments. In addition, there were no obvious changes in the levels of AST, ALT, ALP, and CREA, indicating that PIH-NO did not damage the liver or kidney (Figure S25 and S26).

### Reverse immunosuppression of TME *in vivo*

**Figure 8:**
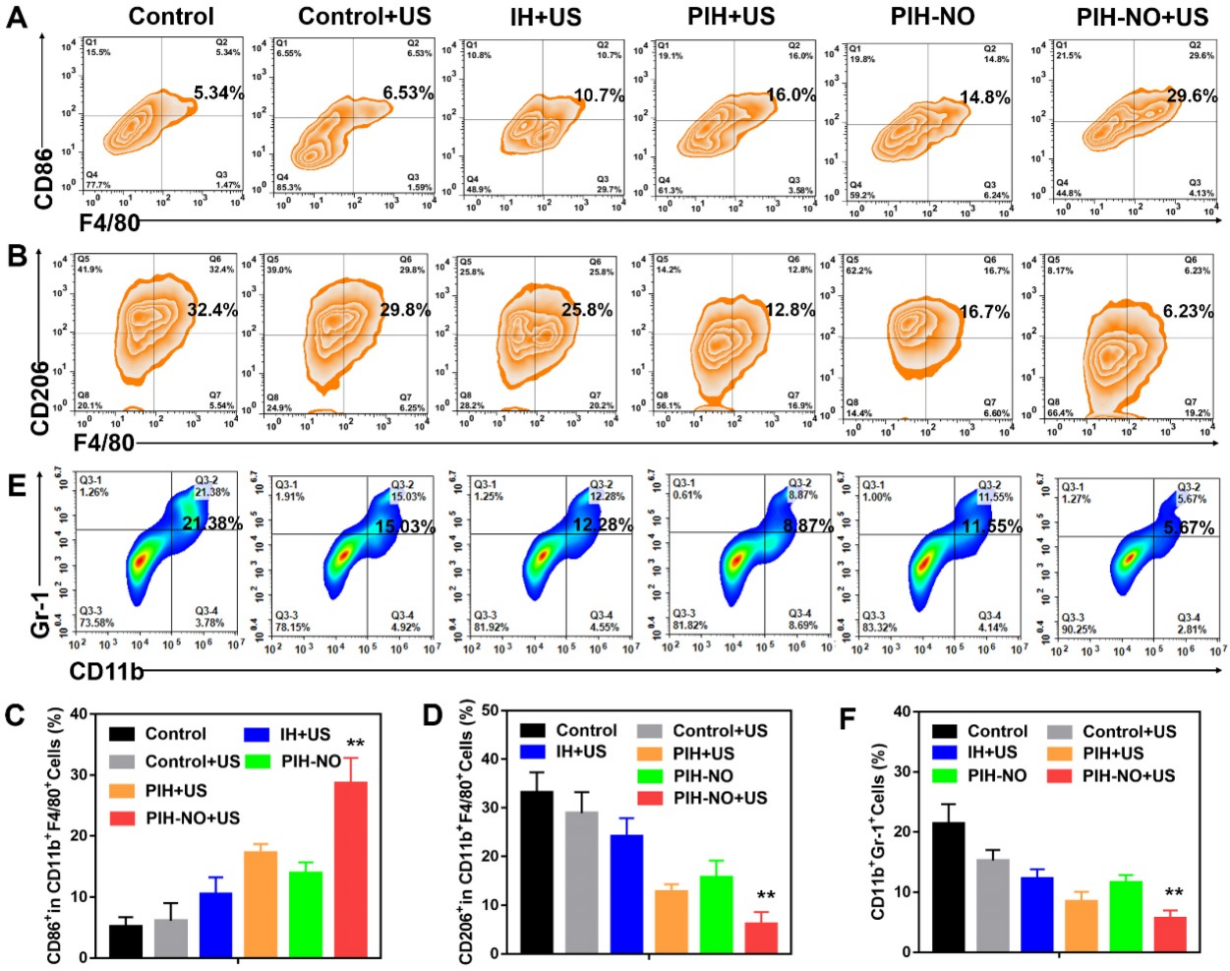
Reverse immunosuppression of tumor microenvironment *in vivo*. (A, B) Flow cytometry analysis of M1 (CD11c^+^F4/80^+^CD86^+^) and M2 (CD11c^+^F4/80^+^CD206^+^) in treated tumors. (C, D) The proportion of M1 and M2 according to flow cytometry analysis. (E, F) Representative flow cytometry plots and percentages of MDSCs (CD11b^+^Gr-1^+^) in treated tumors. **p <0.01 (PIH-NO with US vs. other treatments). Data are expressed as means ± SD (n = 5).

The hypoxic TME induces immunosuppression by activating M2 macrophages and recruiting MDCSs. Previous studies have shown that the attraction of MDSCs to tumors is related to the deteriorated hypoxic microenvironment 53. Oxygen supply and NO release by nanoparticles at the tumor site could reeducate M2 macrophages to become M1 macrophages and attenuate MDSCs to reverse immunosuppression, which increases tumor immunotherapy. After various treatments, the tumors were collected on day 3 and analyzed by flow cytometry. As shown in Figure 8A-D, the number of M1 macrophages (CD11b^+^F4/80^+^CD86^+^) were significantly increased after PIH-NO with US irradiation (29.6%; nearly 2-fold that of the PIH-NO group (14.8%)). This increase was due to the oxygen supply for SDT and NO release upon US irradiation. Additionally, the IH and PIH with US irradiation treatments showed higher numbers of M1 macrophages than that of the control with US irradiation groups (10.7% and 16.0% vs. 6.3%) due to ROS generation accelerating the formation of M1 macrophages 54. Compared with ROS, RNS are more active and toxic. Previous studies have confirmed that RNS could increase M1 repolarization by NF-κB activation and further the inflammatory response 30, 55. The percentages of M2 macrophages (CD11b^+^F4/80^+^CD206^+^) were significantly decreased after PIH-NO treatment, which is the opposite results obtained for M1 macrophages. These results indicated that PIH-NO with oxygen and NO supply could significantly reeducate M2 macrophages to M1 macrophages for immunotherapy, which is similar to the results in previous studies.

The attraction of MDSCs to tumors was characterized by the positive expression of both CD11b and Gr1 using flow cytometry. As shown in Figure 8E and F, without any treatment, the percentage of MDSCs (CD11b^+^Gr-1^+^) in tumor was 21.38% higher than that of other groups. Upon US irradiation, the numbers of MDSCs were significantly reduced in all groups due to the ROS generation and hypoxia relief. The percentage of MDSCs in the tumors after PIH-NO with US treatment significantly decreased to 5.67%, which is nearly a 4-fold decrease relative to the control group. These results revealed that PIH-NO triggered by US released NO and oxygen for SDT enhancement, reversing immunosuppression of the TME to activate an antitumor immune response.

### The amplification of ICD and provoked immune response of PIH-NO *in vivo*

**Figure 9:**
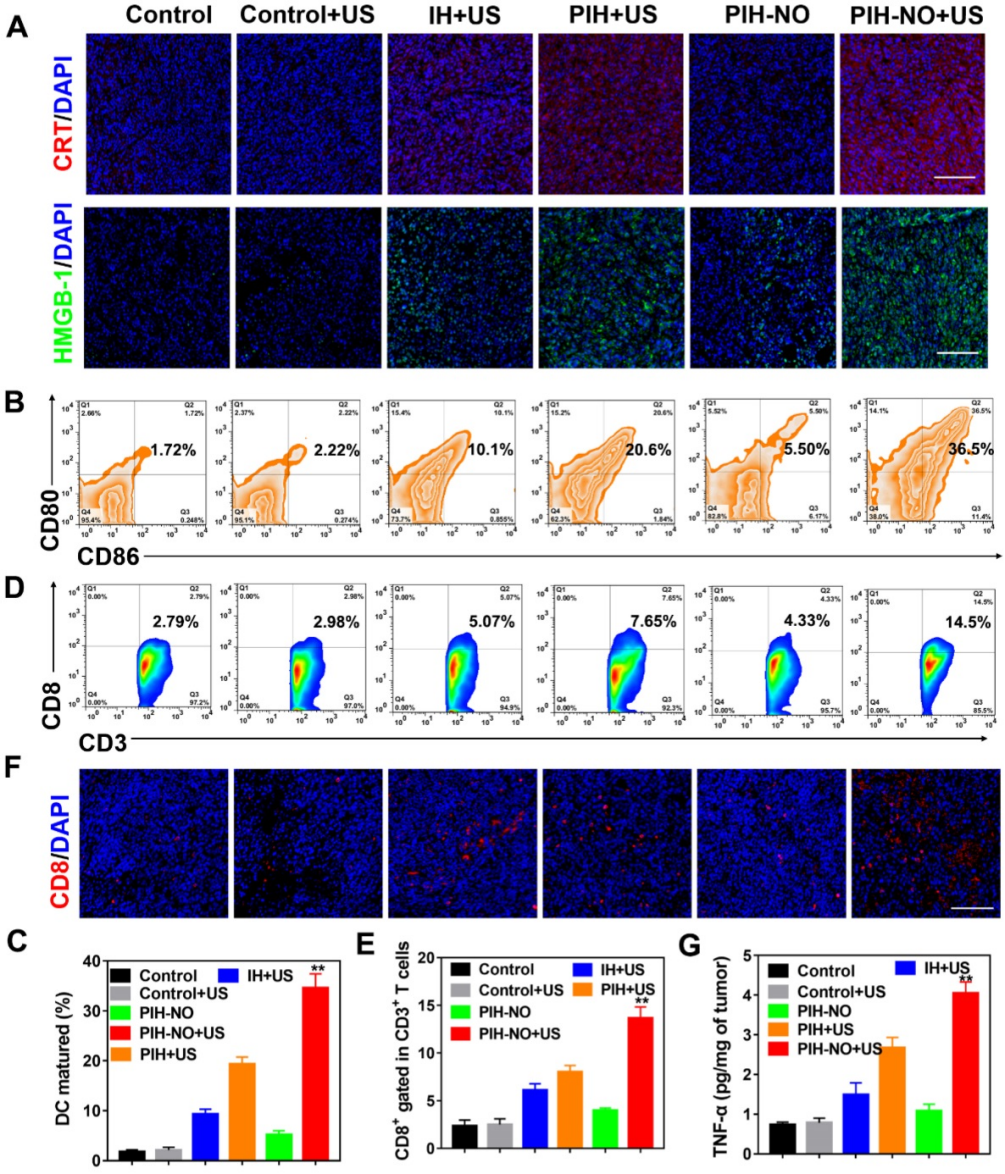
Anti-tumor immune response after PIH-NO treatment. (A) CRT and HMGB-1 immunofluorescence staining of tumor slices after various treatments. The scale bar is 100 μm. (B, C) Representative flow cytometry plots and percentages of DCs (CD11b^+^CD86^+^CD80^+^) in tumor-draining lymph nodes for DC maturation *in vivo*. (D-F) Flow cytometry analysis, percentages, and immunofluorescence staining of CD8^+^ T cells (gated CD3^+^) in tumors. The scale bar is 100 μm. (G) TNF-α expression determined by ELISA. **p <0.01 (PIH-NO with US vs. other treatments). Data are expressed as means ± SD (n = 5).

Upon reversal of immunosuppression, the enhanced tumor-specific immune response was further evaluated by the population of infiltrating immune cells (CLTs, CD8^+^). The ICD markers CRT and HMGB-1 in tumors were first determined by immunofluorescence staining. As shown in Figure 9A and S27, fluorescence signals were observed in the groups treated with US, indicating that the generation of ROS from SDT could induce ICD to activate the immune response. Bright red fluorescence was observed in the PIH-NO plus US irradiation group, and this value was approximately 2.4-fold greater than that of the PIH with US treatment group (MFI, 965 vs. 410). The expression levels of HMGB-1 after various treatments were similar to those of CRT exposure in dying tumor cells (Figure S28). These results indicated that the US-triggered NO and oxygen release could significantly enhance SDT to amplify ICD.

The release of CRT and HMGB-1 could promote DC maturation and provoke a tumor immune response. Owing to ICD-induction by PIH-NO, the maturation of DCs (CD11c^+^CD80^+^CD86^+^) in lymph nodes was analyzed by flow cytometry at three days after various treatments. Owing to the enhancement in SDT and NO gas therapy of PIH-NO with US irradiation, the percent DCs maturation was 36.5%, which was nearly 3.6-fold and 1.5-fold that of IH (10.1%) and PIH (20.6%) with US irradiation, respectively (Figure 9B and C). These results indicated that PIH-NO could amplify ICD in tumor cells and promote DC maturation for immunotherapy.

The increased number of mature DCs enhanced antigen presentation to induce a tumor-specific immune response by enhanced cytotoxic T lymphocytes (CTLs, CD8^+^). Treated tumors were collected on day 7 and analyzed by flow cytometry. Figure 9D and E showed that the percentage of CTLs (CD3^+^CD8^+^) in the PIH-NO plus US treatment group significantly increased to 14.5%, which was higher than that in the IH (5.07%) and PIH (7.65%) with US treatment groups. This result was further confirmed by immunofluorescence staining of CD8^+^ cells at the tumor site. Significantly more CD8^+^ T cells (red fluorescence) were observed in the PIH-NO with US treatment, which is consistent with the flow cytometry analysis (Figure 9F). These results indicated that the enhanced SDT and NO produced by PIH-NO with US treatment could increase T cell infiltration for cancer immunotherapy. The expression of tumor necrosis factor-α (TNF-α) was significantly increased in the PIH-NO with US irradiation due to the better activation of CD8^+^ T cells. Immunofluorescence staining of IFN-γ was also performed to confirm the enhanced CTLs. As shown in Figure S29, bright green fluorescence was observed in the PIH-NO with US irradiation, which was more intense than that in other groups and indicated an enhanced immune response. These collective results demonstrated that PIH-NO could amplify ICD and activate an antitumor immune response for tumor and metastasis inhibition.

## Conclusion

In summary, we designed an US-responsive nanoparticle to deliver oxygen and NO. This system can reverse immunosuppression and enhance SDT to activate an antitumor immune response. Based on the mitochondria-targeting properties of IR780, PIH-NO can preferentially accumulate and be retained in the mitochondria. Upon US irradiation, the system displays a burst oxygen release of oxygen to overcome hypoxia and increase ^1^O_2_ generation for SDT enhancement. In addition, this system can also be activated by US to release NO, further generating stronger ONOO- for cancer treatment *in vitro*. After intravenous injection into tumor-bearing mice, the system showed a significant enhancement in the accumulation at the tumor site and increased blood perfusion due to the oxygen and NO release, which in turn reduced tumor hypoxia. With US treatment, PIH-NO significantly suppressed tumor growth and inhibited lung metastasis due to the combination of enhanced SDT and NO gas therapy. More importantly, PIH-NO remodeled the immunosuppressive microenvironment by reeducating M2 macrophages to M1 phenotype and decreasing MDSC recruitment. Extensive *in vitro* and *iv vivo* evaluations demonstrated that this approach could induce and amplify ICD to promote DCs maturation, thus increasing the population of infiltrating immune cells to provoke an antitumor immune response. This study provides a promising strategy of gas delivery to enhance the therapeutic efficacy of SDT and activate an antitumor immune response.

## Supplementary Material

Supplementary figures and table.

## References

[1] Gong F, Cheng L, Yang N, Gong Y, Ni Y, Bai S (2020). Preparation of TiH 1.924 nanodots by liquid-phase exfoliation for enhanced sonodynamic cancer therapy. Nat Commun.

[2] Canavese G, Ancona A, Racca L, Canta M, Dumontel B, Barbaresco F (2018). Nanoparticle-assisted ultrasound: A special focus on sonodynamic therapy against cancer. Chem Eng J.

[3] Qian X, Zheng Y, Chen Y (2016). Micro/nanoparticle-augmented sonodynamic therapy (SDT): Breaking the depth shallow of photoactivation. Adv Mater.

[4] Ludin A, Zon LI (2017). The dark side of PD-1 receptor inhibition. Nature.

[5] Zhao H, Zhao B, Li L, Ding K, Xiao H, Zheng C (2020). Biomimetic decoy inhibits tumor growth and lung metastasis by reversing the drawbacks of sonodynamic therapy. Adv Healthc Mater.

[6] Yang X, Yang Y, Gao F, Wei J-J, Qian C-G, Sun M-J (2019). Biomimetic hybrid nanozymes with self-supplied H+ and accelerated O2 generation for enhanced starvation and photodynamic therapy against hypoxic tumors. Nano Lett.

[7] Fu J, Li T, Zhu Y, Hao Y (2019). Ultrasound-activated oxygen and ROS generation nanosystem systematically modulates tumor microenvironment and sensitizes sonodynamic therapy for hypoxic solid tumors. Adv Funct Mater.

[8] Wang Z, Zhang Y, Ju E, Liu Z, Cao F, Chen Z (2018). Biomimetic nanoflowers by self-assembly of nanozymes to induce intracellular oxidative damage against hypoxic tumors. Nat Commun.

[9] Zhu P, Chen Y, Shi J (2018). Nanoenzyme-augmented cancer sonodynamic therapy by catalytic tumor oxygenation. ACS Nano.

[10] Yang B, Chen Y, Shi J (2019). Nanocatalytic medicine. Adv Mater.

[11] Jiang W, Zhang Z, Wang Q, Dou J, Zhao Y, Ma Y (2019). Tumor reoxygenation and blood perfusion enhanced photodynamic therapy using ultrathin graphdiyne oxide nanosheets. Nano Lett.

[12] Wang L, Niu M, Zheng C, Zhao H, Niu X, Li L (2018). A core-shell nanoplatform for synergistic enhanced sonodynamic therapy of hypoxic tumor via cascaded strategy. Adv Healthc Mater.

[13] Gao M, Liang C, Song X, Chen Q, Jin Q, Wang C (2017). Erythrocyte-Membrane-Enveloped Perfluorocarbon as Nanoscale Artificial Red Blood Cells to Relieve Tumor Hypoxia and Enhance Cancer Radiotherapy. Adv Mater.

[14] Zhou Z, Zhang B, Wang S, Zai W, Yuan A, Hu Y (2018). Perfluorocarbon Nanoparticles Mediated Platelet Blocking Disrupt Vascular Barriers to Improve the Efficacy of Oxygen-Sensitive Antitumor Drugs. Small.

[15] Song G, Ji C, Liang C, Song X, Yi X, Dong Z (2017). TaOx decorated perfluorocarbon nanodroplets as oxygen reservoirs to overcome tumor hypoxia and enhance cancer radiotherapy. Biomaterials.

[16] Song X, Feng L, Liang C, Yang K, Liu Z (2016). Ultrasound triggered tumor oxygenation with oxygen-shuttle nanoperfluorocarbon to overcome hypoxia-associated resistance in cancer therapies. Nano Lett.

[17] Chen Q, He Y, Wang Y, Li C, Zhang Y, Guo Q (2020). Penetrable Nanoplatform for “Cold” Tumor Immune Microenvironment Reeducation. Adv Sci.

[18] Kumar V, Patel S, Tcyganov E, Gabrilovich DI (2016). The nature of myeloid-derived suppressor cells in the tumor microenvironment. Trends Immunol.

[19] Chen B, Gao A, Tu B, Wang Y, Yu X, Wang Y (2020). Metabolic modulation via mTOR pathway and anti-angiogenesis remodels tumor microenvironment using PD-L1-targeting codelivery. Biomaterials.

[20] Li L, Zhen M, Wang H, Sun Z, Jia W, Zhao Z (2020). Functional Gadofullerene Nanoparticles Trigger Robust Cancer Immunotherapy Based on Rebuilding an Immunosuppressive Tumor Microenvironment. Nano Lett.

[21] Phuengkham H, Ren L, Shin IW, Lim YT (2019). Nanoengineered immune niches for reprogramming the immunosuppressive tumor microenvironment and enhancing cancer immunotherapy. Adv Mater.

[22] Xu Y, Liu J, Liu Z, Ren H, Yong J, Li W (2020). Blockade of Platelets Using Tumor-Specific NO-Releasing Nanoparticles Prevents Tumor Metastasis and Reverses Tumor Immunosuppression. ACS Nano.

[23] Zhou X, Meng Z, She J, Zhang Y, Yi X, Zhou H (2020). Near-infrared light-responsive nitric oxide delivery platform for enhanced radioimmunotherapy. Nanomicro Lett.

[24] Sung Y-C, Jin P-R, Chu L-A, Hsu F-F, Wang M-R, Chang C-C (2019). Delivery of nitric oxide with a nanocarrier promotes tumour vessel normalization and potentiates anti-cancer therapies. Nat Nanotechnol.

[25] Liu F, Gong S, Shen M, He T, Liang X, Shu Y (2021). A glutathione-activatable nanoplatform for enhanced photodynamic therapy with simultaneous hypoxia relief and glutathione depletion. Chem Eng J.

[26] Ramesh A, Kumar S, Brouillard A, Nandi D, Kulkarni A (2020). A Nitric Oxide (NO) Nanoreporter for Noninvasive Real-Time Imaging of Macrophage Immunotherapy. Adv Mater.

[27] Tian L, Wang Y, Sun L, Xu J, Chao Y, Yang K (2019). Cerenkov luminescence-induced NO release from 32P-labeled ZnFe (CN) 5NO nanosheets to enhance radioisotope-immunotherapy. Matter.

[28] Lin YJ, Chen CC, Nguyen D, Su HR, Lin KJ, Chen HL (2020). Biomimetic Engineering of a Scavenger-Free Nitric Oxide-Generating/Delivering System to Enhance Radiation Therapy. Small.

[29] Yuan Z, Lin C, He Y, Tao B, Chen M, Zhang J (2020). Near-Infrared light-triggered nitric-oxide-enhanced photodynamic therapy and low-temperature photothermal therapy for biofilm elimination. ACS Nano.

[30] Feng Q, Li Y, Wang N, Hao Y, Chang J, Wang Z (2020). A biomimetic nanogenerator of reactive nitrogen species based on battlefield transfer strategy for enhanced immunotherapy. Small.

[31] Wei G, Yang G, Wei B, Wang Y, Zhou S (2019). Near-infrared light switching nitric oxide nanoemitter for triple-combination therapy of multidrug resistant cancer. Acta Biomater.

[32] Xu Y, Ren H, Liu J, Wang Y, Meng Z, He Z (2019). A switchable NO-releasing nanomedicine for enhanced cancer therapy and inhibition of metastasis. Nanoscale.

[33] Zhang K, Xu H, Jia X, Chen Y, Ma M, Sun L (2016). Ultrasound-triggered nitric oxide release platform based on energy transformation for targeted inhibition of pancreatic tumor. ACS Nano.

[34] An J, Hu Y-G, Li C, Hou X-L, Cheng K, Zhang B (2020). A pH/ultrasound dual-response biomimetic nanoplatform for nitric oxide gas-sonodynamic combined therapy and repeated ultrasound for relieving hypoxia. Biomaterials.

[35] Zhang W, Hu X, Shen Q, Xing D (2019). Mitochondria-specific drug release and reactive oxygen species burst induced by polyprodrug nanoreactors can enhance chemotherapy. Nat Commun.

[36] Wang Y, Shim MS, Levinson NS, Sung HW, Xia Y (2014). Stimuli-responsive materials for controlled release of theranostic agents. Adv Funct Mater.

[37] Murphy MP, Hartley RC (2018). Mitochondria as a therapeutic target for common pathologies. Nat Rev Drug Discov.

[38] Xu J, Zeng F, Wu H, Hu C, Yu C, Wu S (2014). Preparation of a mitochondria-targeted and NO-releasing nanoplatform and its enhanced pro-apoptotic effect on cancer cells. Small.

[39] Chen X, Li Y, Li S, Gao M, Ren L, Tang BZ (2018). Mitochondria-and lysosomes-targeted synergistic chemo-photodynamic therapy associated with self-monitoring by dual light-up fluorescence. Adv Funct Mater.

[40] Zhang L, Wang D, Yang K, Sheng D, Tan B, Wang Z (2018). Mitochondria-Targeted Artificial “Nano-RBCs” for Amplified Synergistic Cancer Phototherapy by a Single NIR Irradiation. Adv Sci.

[41] Li Y, Zhou Q, Deng Z, Pan M, Liu X, Wu J (2016). IR-780 dye as a sonosensitizer for sonodynamic therapy of breast tumor. Sci Rep.

[42] Li W, Yong J, Xu Y, Wang Y, Zhang Y, Ren H (2019). Glutathione depletion and dual-model oxygen balance disruption for photodynamic therapy enhancement. Colloids Surf B Biointerfaces.

[43] Wang Y, Liu T, Zhang E, Luo S, Tan X, Shi C (2014). Preferential accumulation of the near infrared heptamethine dye IR-780 in the mitochondria of drug-resistant lung cancer cells. Biomaterials.

[44] Zhang L, Yi H, Song J, Huang J, Yang K, Tan B (2019). Mitochondria-targeted and ultrasound-activated nanodroplets for enhanced deep-penetration sonodynamic cancer therapy. ACS Appl Mater Interfaces.

[45] Jiang C, Cheng H, Yuan A, Tang X, Wu J, Hu Y (2015). Hydrophobic IR780 encapsulated in biodegradable human serum albumin nanoparticles for photothermal and photodynamic therapy. Acta Biomater.

[46] Hassanin I, Elzoghby A (2020). Albumin-based nanoparticles: a promising strategy to overcome cancer drug resistance. Cancer Drug Resist.

[47] Zhang C, Long L, Shi C (2018). Mitochondria-targeting IR-780 dye and its derivatives: synthesis, mechanisms of action, and theranostic applications. Adv Ther.

[48] Yue W, Chen L, Yu L, Zhou B, Yin H, Ren W (2019). Checkpoint blockade and nanosonosensitizer-augmented noninvasive sonodynamic therapy combination reduces tumour growth and metastases in mice. Nat Commun.

[49] Wang Y, Liu Z, Wang H, Meng Z, Wang Y, Miao W (2019). Starvation-amplified CO generation for enhanced cancer therapy via an erythrocyte membrane-biomimetic gas nanofactory. Acta Biomater.

[50] Islam W, Fang J, Imamura T, Etrych T, Subr V, Ulbrich K (2018). Augmentation of the enhanced permeability and retention effect with nitric oxide-generating agents improves the therapeutic effects of nanomedicines. Mol Cancer Ther.

[51] Dong X, Liu H-J, Feng H-Y, Yang S-C, Liu X-L, Lai X (2019). Enhanced drug delivery by nanoscale integration of a nitric oxide donor to induce tumor collagen depletion. Nano Lett.

[52] Wang C, Li Y, Shi X, Zhou J, Zhou L, Wei S (2018). Use of an NIR-light-responsive CO nanodonor to improve the EPR effect in photothermal cancer treatment. Chem Commun.

[53] Zhang C, Xia D, Liu J, Huo D, Jiang X, Hu Y (2020). Bypassing the Immunosuppression of Myeloid-Derived Suppressor Cells by Reversing Tumor Hypoxia Using a Platelet-Inspired Platform. Adv Funct Mater.

[54] Zhang Q, Bao C, Cai X, Jin L, Sun L, Lang Y (2018). Sonodynamic therapy-assisted immunotherapy: A novel modality for cancer treatment. Cancer Sci.

[55] Szabó C, Ischiropoulos H, Radi R (2007). Peroxynitrite: biochemistry, pathophysiology and development of therapeutics. Nat Rev Drug Discov.

