# Supplementary material for: Mitochondria-targeted and ultrasound-responsive nanoparticles for oxygen and nitric oxide codelivery to reverse immunosuppression and enhance sonodynamic therapy for immune activation
Source: Theranostics. 2021 Jul 25;11(17):8587–604. doi: 10.7150/thno.62572 (PMC8344010; doi:10.7150/thno.62572)
Supplement: Supplementary file 1 — Supplementary figures and table. [file thnov11p8587s1.pdf]

Supporting information

Mitochondria-targeted and Ultrasound-responsive Nanoparticles for Oxygen and Nitric Oxide Co-delivery to Reverse Immunosuppression and Enhance Sonodynamic Therapy for Immune Activation

Changwei Ji[1,#], Jingxing Si[2,3,#], Yan Xu[4], Wenjing Zhang[4], Yaqian Yang[4], Xin He[1], Hao Xu[4], Xiaozhou Mou[2,3]*, Hao Ren[4]*, Hongqian Guo[1]*

Affiliations:

1. Department of Urology, Drum Tower Hospital, Medical school of Nanjing University, Institute of Urology, Nanjing University, Nanjing 210008, Jiangsu, China.
2. Key Laboratory of Tumor Molecular Diagnosis and Individualized Medicine of Zhejiang Province, Zhejiang Provincial People's Hospital, People's Hospital of Hangzhou Medical College, Hangzhou 310014, Zhejiang, China.
3. Clinical Research Institute, Zhejiang Provincial People's Hospital, People's Hospital of Hangzhou Medical College, Hangzhou 310014, Zhejiang, China.
4. School of Pharmaceutical Science, Nanjing Tech University, Nanjing 211816, Jiangsu, China.

*Author for correspondence:
Dr. Xiaozhou Mou, PhD
Email: mouxz@zju.edu.cn
Prof. Hao Ren, PhD.
E-mail: hren@njtech.edu.cn
Dr. Hongqian Guo, Ph.D.
E-mail: dr.ghq@nju.edu.cn

[#]These authors contributed equally to the article

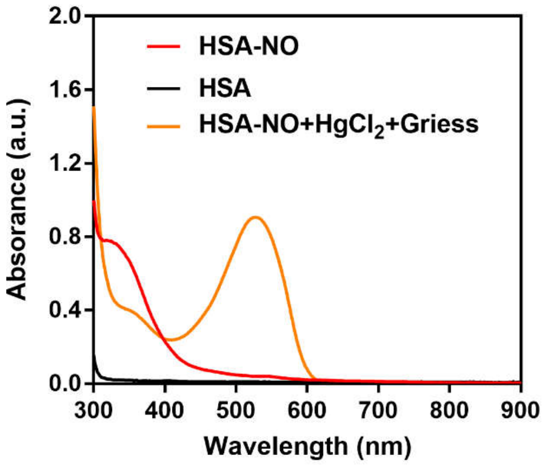

Figure S1. UV-vis absorption spectra of HSA, HSA-NO and HSA-SNO with Griess assay and $HgCl_2$

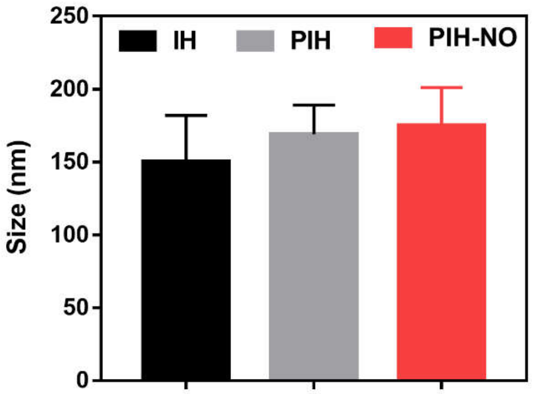

Figure S2. The average size of IH, PIH and PIH-NO determined by DLS.

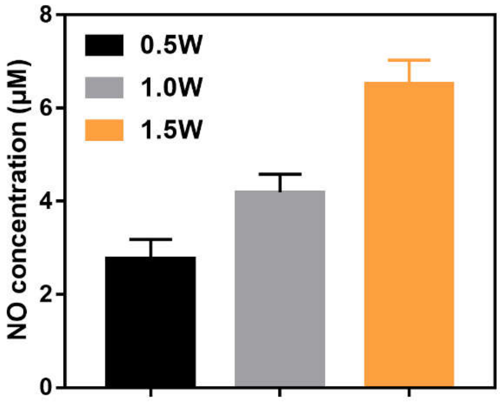

Figure S3. The release of NO with different ultrasound treatment (0.5, 1.0, and 1.5W/cm^2) for 3min.

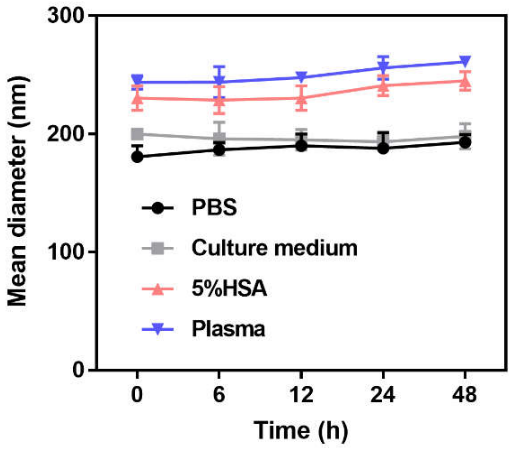

Figure S4. The stability of PIH-NO in PBS, culture medium, 5% HSA and plasma within 48 h at room temperature.

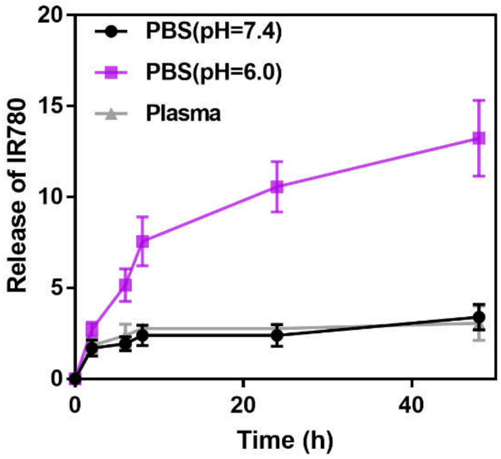

Figure S5. The release of IR780 in PIH-NO at PBS (pH=7.4 and 6.0) and plasma within 48 h.

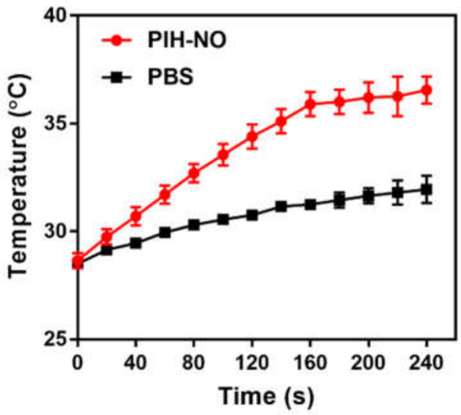

Figure S6. The increased temperature of PIH-NO (IR780, 40μg/mL) and PBS with US irradiation time. Data are expressed as means ± SD (n = 3).

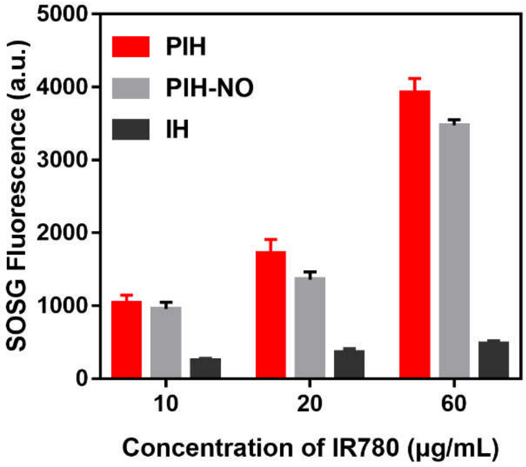

Figure S7. The $^1O_2$ generation in PIH, PIH-NO and IH with different concentrations after US treatment (1.0 W/cm^2) for 3 min.

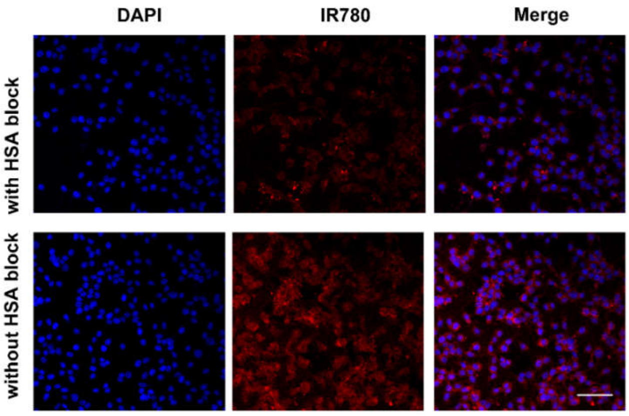

Figure S8. The cellular uptake of PIH-NO after with or without HSA block. The scale bar is 100 μm.

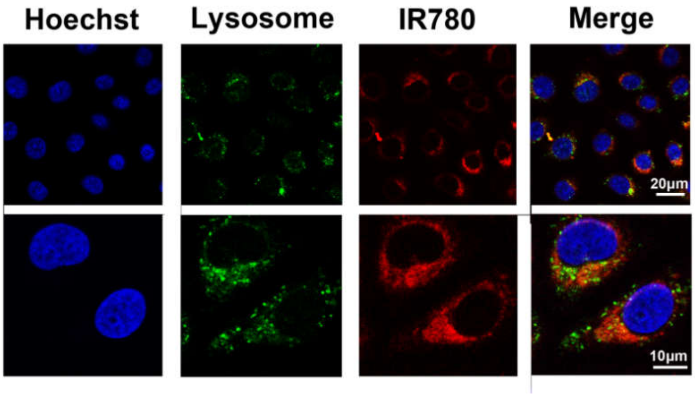

Figure S9. Representative fluorescence confocal images of lysosome and IR780 after PIH-NO incubated with 4T1 cells for 3 h.

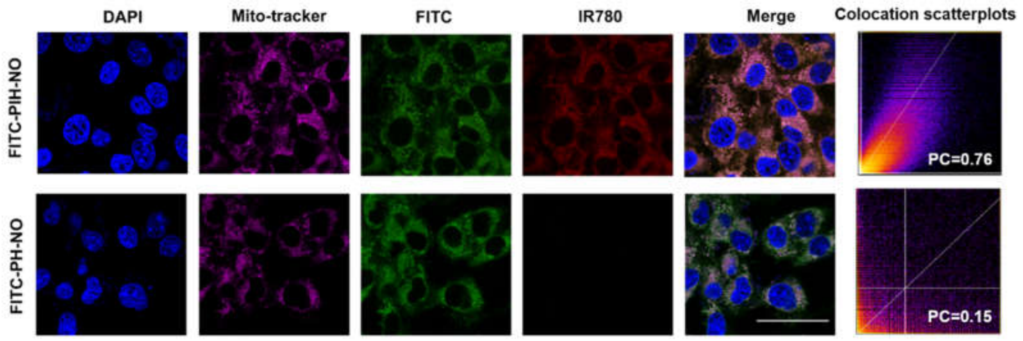

Figure S10. The cellular uptake and mito-targeted properties of FITC labeled PIH-NO and PH-NO after incubation with 4T1 cells. The scale bar is 50 μm.

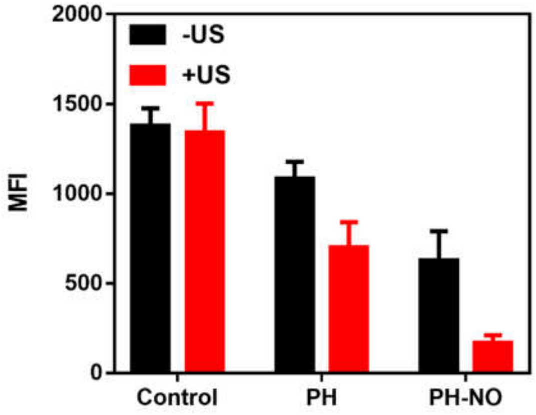

Figure S11. The mean fluorescence intensity of hypoxia in tumor cells after incubation with saline, PH and PH-NO in hypoxic condition.

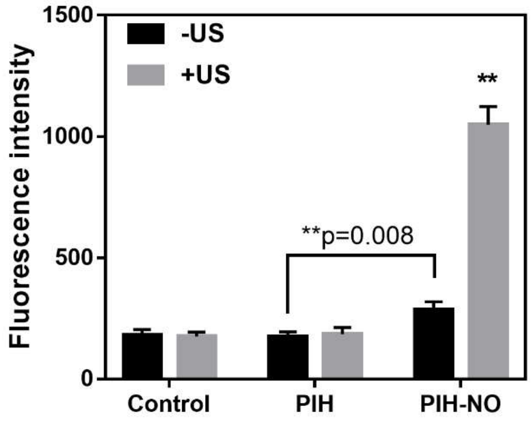

Figure S12. Quantification of intracellular NO release by fluorescence microplate in various treatments. **p <0.01 (PIH-NO with US vs. other treatments and PIH-NO without US vs. PIH without US).

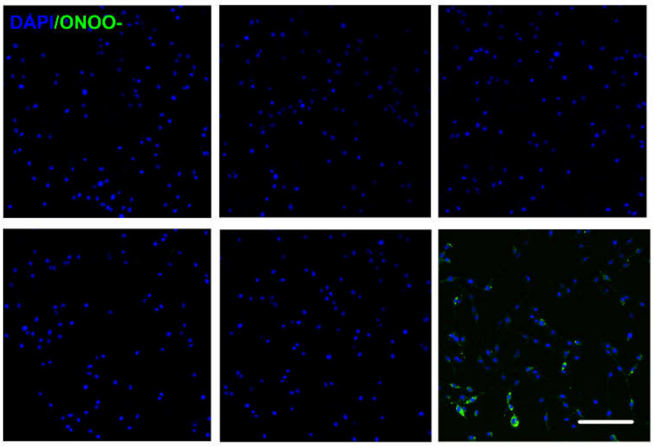

Figure S13. The intracellular generation of ONOO- after various treatments.

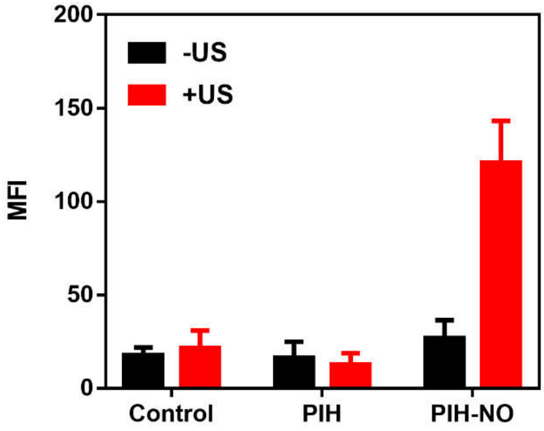

Figure S14. The mean fluorescence intensity of ONOO- in tumor cells calculated based on DHR staining.

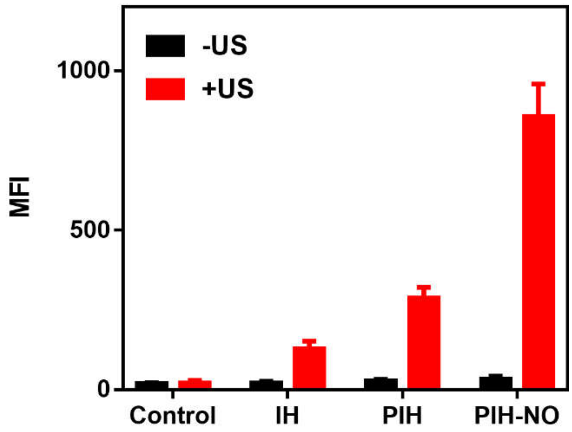

Figure S15. The mean fluorescence intensity of LPO in mitochondria calculated based on MitoPeDPP staining.

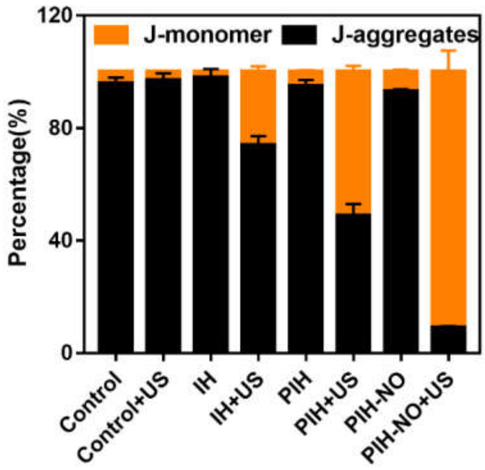

Figure S16. The percentage of J-monomer and J-aggregates based on JC-1 staining after various treatments.

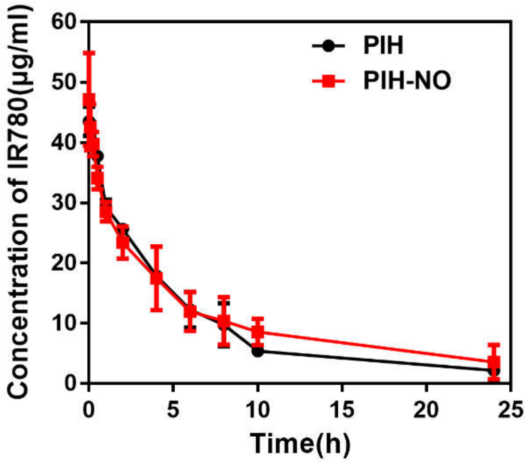

Figure S17. *In vivo* pharmacokinetic curves of PIH-NO and PIH within 24 h.

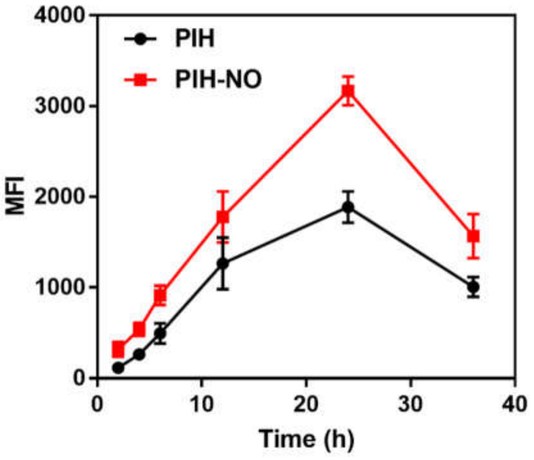

Figure S18. The time-dependent changes of mean fluorescence intensity of IR780 after intravenous injection into tumor bearing

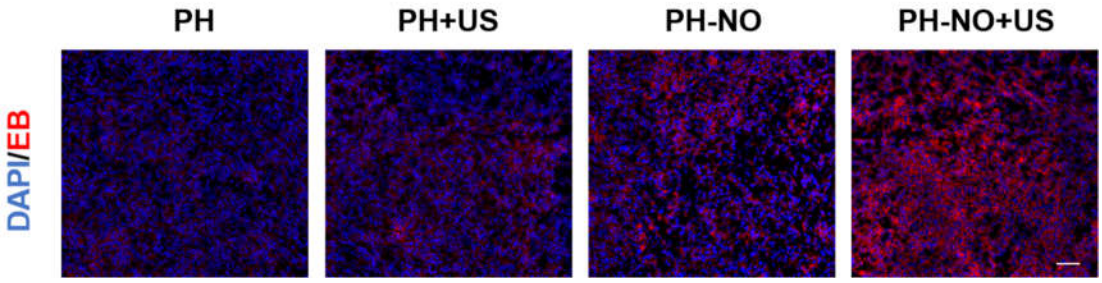

Figure S19. Representative fluorescence images of Evans blue in tumor frozen slice. The scale bar is 50 μm.

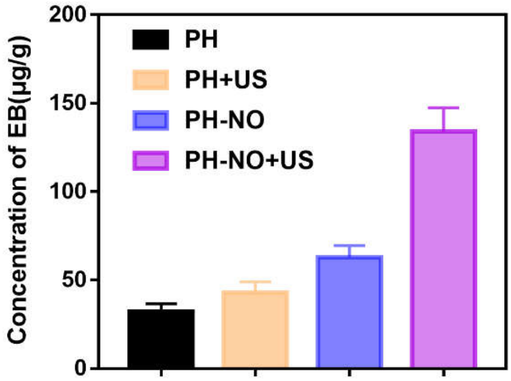

Figure S20. The concentration of Evans blue after different treatments (n = 3).

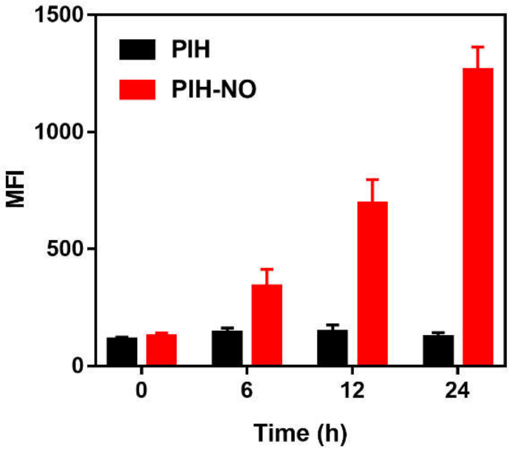

Figure S21. The time-dependent changes of mean fluorescence intensity of blood perfusion determined by doppler flowmeter after intravenous injection into tumor bearing

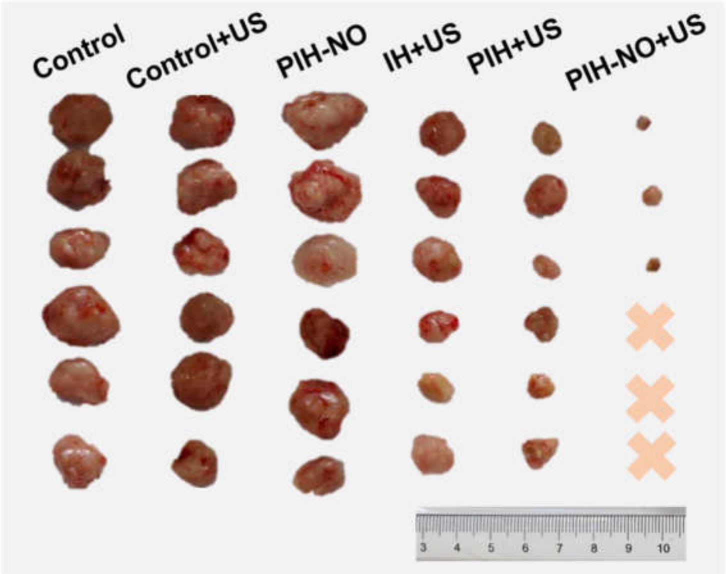

Figure S22. The photographs of tumor after various treatments at day 14.

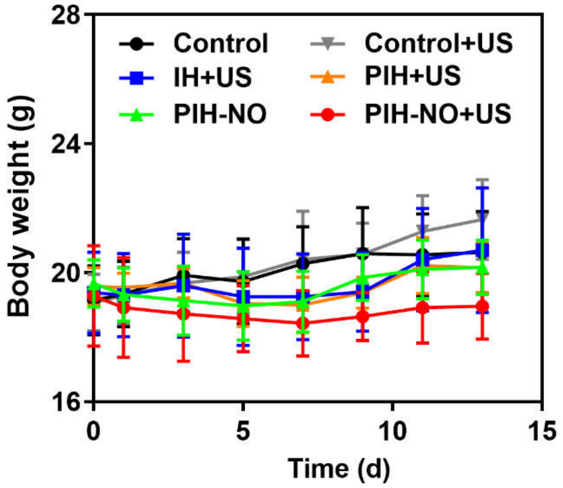

Figure S23. The changes of body weight during treatments.

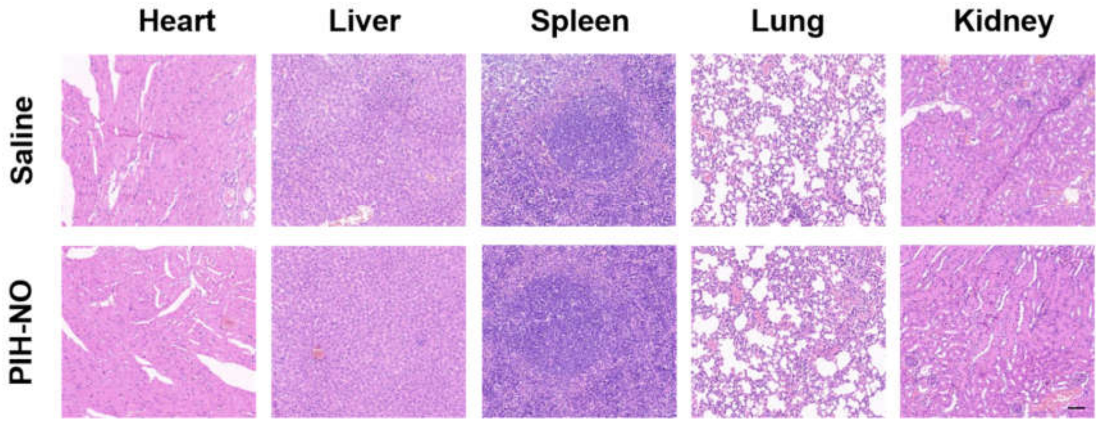

Figure S24. The H&E staining of major organs after intravenous injection of PIH-NO and saline. The scale bar is 100 μm.

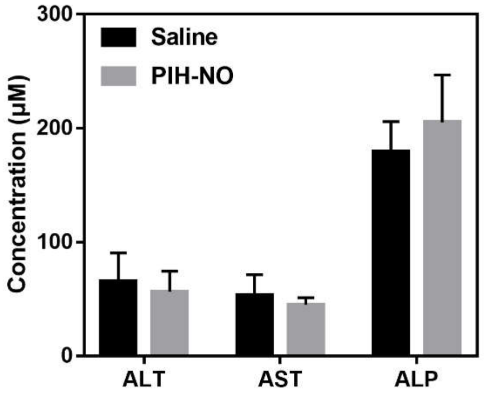

Figure S25. The liver function determined by AST, ALT and ALP after intravenous injection of PIH-NO and saline

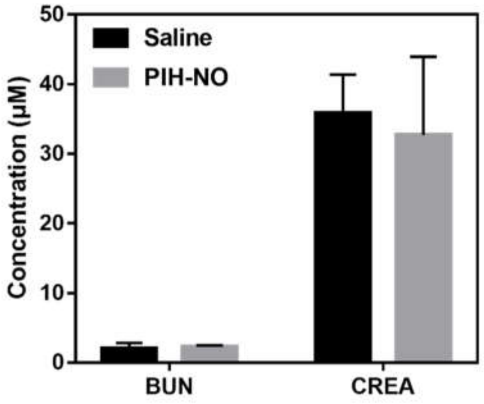

Figure S26. The kidney function determined by BUN and CREA after intravenous injection of PIH-NO and saline

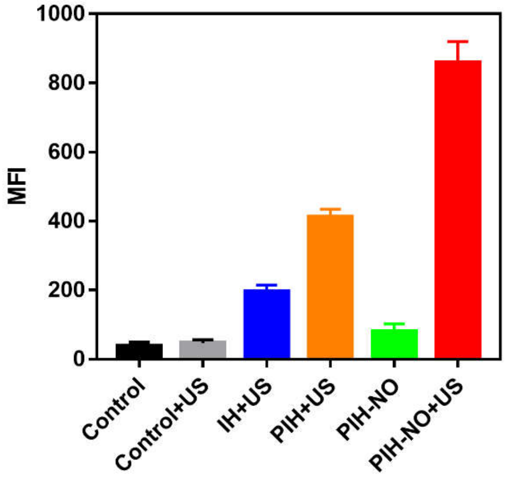

Figure S27. The mean fluorescence intensity of CRT exposure in tumor after various treatments

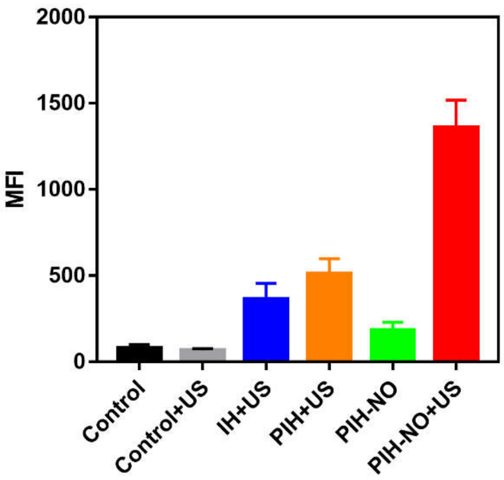

Figure S28. The mean fluorescence intensity of HMGB-1 staining in tumor after various treatments

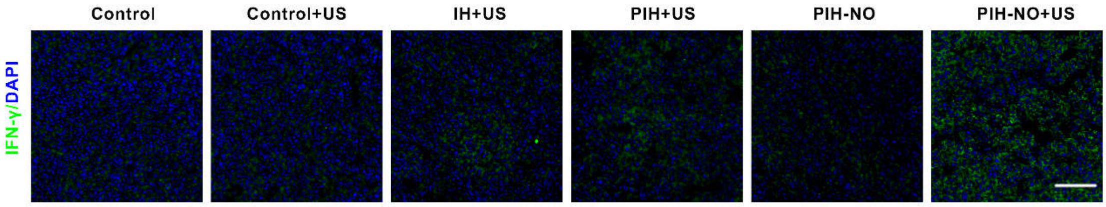

Figure S29. Representative immunofluorescence images of IFN-γ of tumor slices after various treatments. The scale bar is 200μm.

Table S1. Pharmacokinetic parameters of IR780 in mice plasma after intravenous injection with PIH and PIH-NO.

Group	t1/2(h)	AUC(mg/L*h)	MRT(h)	Cmax (mg/L)
PIH	9.26±0.62	217.02±10.12	9.94±0.72	44.19±4.27
PIH-NO	8.79±1.14	232.16±19.37	8.62±0.91	47.63±6.19

The elimination half lifetimes were calculated by DAS 3.2 software (Mathematical Pharmacology Professional Committee of China, Shanghai, China).
